# Cytokines: From Clinical Significance to Quantification

**DOI:** 10.1002/advs.202004433

**Published:** 2021-06-10

**Authors:** Chao Liu, Dewei Chu, Kourosh Kalantar‐Zadeh, Jacob George, Howard A. Young, Guozhen Liu

**Affiliations:** ^1^ School of Materials Science and Engineering University of New South Wales Sydney NSW 2052 Australia; ^2^ School of Chemical Engineering University of New South Wales Sydney NSW 2052 Australia; ^3^ Storr Liver Centre Westmead Institute of Medical Research University of Sydney and Department of Gastroenterology and Hepatology Westmead Hospital Westmead NSW 2145 Australia; ^4^ Laboratory of Cancer Immunometabolism Center for Cancer Research National Cancer Institute at Frederick Frederick MD 21702 USA; ^5^ School of Life and Health Sciences The Chinese University of Hong Kong Shenzhen 518172 P. R. China; ^6^ Graduate School of Biomedical Engineering University of New South Wales Sydney NSW 2052 Australia

**Keywords:** biosensors, clinical significance, cytokines, in vitro and in vivo assays, quantification

## Abstract

Cytokines are critical mediators that oversee and regulate immune and inflammatory responses via complex networks and serve as biomarkers for many diseases. Quantification of cytokines has significant value in both clinical medicine and biology as the levels provide insights into physiological and pathological processes and can be used to aid diagnosis and treatment. Cytokines and their clinical significance are introduced from the perspective of their pro‐ and anti‐inflammatory effects. Factors affecting cytokines quantification in biological fluids, native levels in different body fluids, sample processing and storage conditions, sensitivity to freeze‐thaw, and soluble cytokine receptors are discussed. In addition, recent advances in in vitro and in vivo assays, biosensors based on different signal outputs and intracellular to extracellular protein expression are summarized. Various quantification platforms for high‐sensitivity and reliable measurement of cytokines in different scenarios are discussed, and commercially available cytokine assays are compared. A discussion of challenges in the development and advancement of technologies for cytokine quantification that aim to achieve real‐time multiplex cytokine analysis for point‐of‐care situations applicable for both biomedical research and clinical practice are discussed.

## Graphical abstract

Cytokines are important cellular signaling molecules and immune system mediators. Abnormal cytokine levels may cause cytokine storm and diseases. Consequently, quantification of cytokines is valuable for diseases diagnosisand therapy. The clinical significance of cytokines, factors affecting cytokine quantification, and advances of cytokine detection are summarized, providing a prospective for real‐time quantification of multiplex cytokines in the clinic.

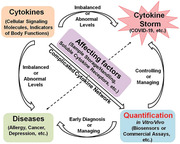

## 1 Introduction

Cytokines are soluble proteins with low molecular weight (≈6–70 kDa), secreted from a variety of cells (lymphocytes, macrophages, natural killer (NK) cells, mast cells, and stromal cells). They participate in the immune response and act as important mediators associated with the communication network of the immune system.^[^ ^1^, ^2^ ^]^ Cytokines are responsible for the dynamic regulation of the maturation, growth and responsiveness of immune cells, and are important determinants of health.^[^ ^3^, ^4^, ^5^ ^]^ A single cytokine may be secreted by different cell types and can act on several cell types, producing multiple biological activities.^[^ ^6^ ^]^ Variation in cytokines levels in various biological fluids such as serum, blood, stool, saliva, and sweat, provides valuable information regarding the diagnosis, stage, and prognosis of various diseases. Abnormal or increased production of cytokines such as during a cytokine storm can lead to organ failure and death. For example, a consensus is that “cytokine storm syndrome” is responsible for the poor prognosis of critical Corona Virus Disease 2019 （COVID‐19) cases.^[^ ^7^, ^8^ ^]^ Consequently, the levels of cytokines are recognized as an essential indicator for evaluating clinical disorders. Accurate quantification of cytokines offers valuable information in the clinical context to monitor the immune status of patients and for adjusting therapies in different diseases, including asthma,^[^ ^9^ ^]^ atherosclerosis,^[^ ^10^ ^]^ cancer,^[^ ^11^ ^]^ depression,^[^ ^12^ ^]^ heart disease,^[^ ^13^ ^]^ Acquired Immune Deficiency Syndrome (AIDS),^[^ ^14^ ^]^ kidney injury,^[^ ^15^ ^]^ sepsis,^[^ ^16^ ^]^ rheumatoid arthritis,^[^ ^17^ ^]^ and other chronic diseases.^[^ ^18^ ^]^

In practice, accurate detection of cytokines is challenging because of their trace amounts (pm range) in the body, their dynamic secretion processes,^[^ ^19^ ^]^ and short half‐lives.^[^ ^20^ ^]^ Cytokines form complex networks that serve to modulate immune processes; different cytokines may have an antagonistic, additive, or synergistic influence on the same biological process. Due to the extreme complexity of the network, measuring cytokines in real time during their response to the surrounding microcellular milieu remains a challenge.^[^ ^21^ ^]^ The most commonly used methods for cytokine quantification are the enzyme linked immunosorbent assay (ELISA)^[^ ^22^ ^]^ and polymerase chain reaction (PCR).^[^ ^23^ ^]^ These methods are reliable but time‐consuming, requiring expensive lab‐based instruments, trained personnel, a long sample preparation time (over 6 h), and high levels of complexity in sample handling. In addition, some approaches may not allow the measurements of multiple cytokines in real time. Consequently, there are unmet demands to develop sensitive, selective, and rapid real time cytokine analysis platforms for quantitative analysis of cytokines from in vitro to in vivo for predicting disease and monitoring the effects of drug for treatments.^[^ ^19^ ^]^ In this regard, biosensors are increasingly attracting attention and being more widely employed.^[^ ^24^, ^25^, ^26^ ^]^ Current investigations are dedicated to developing biosensors such as immunosensors for cytokine detection from intracellular to extracellular regions,^[^ ^19^ ^]^ especially in infectious disease diagnostics^[^ ^27^ ^]^ and drug screening. Aptamers have also garnered interest in biosensing applications due to their small size, reusability as compared to single use antibodies and efficient immobilization at high density.^[^ ^26^ ^]^ A variety of biosensing platforms for quantification of cytokines ranging from sandwich immunosensors^[^ ^28^, ^29^, ^30^ ^]^ to aptasensors,^[^ ^31^, ^32^ ^]^ nanosensors^[^ ^33^, ^34^ ^]^ implantable medical devices,^[^ ^30^, ^35^, ^36^, ^37^ ^]^ point‐of‐care (POC) diagnostics,^[^ ^31^ ^]^ in vivo real‐time monitoring,^[^ ^38^ ^]^ and from intracellular bioimaging^[^ ^39^ ^]^ to extracellular detection^[^ ^40^ ^]^ have been reported.

**Figure 1:**
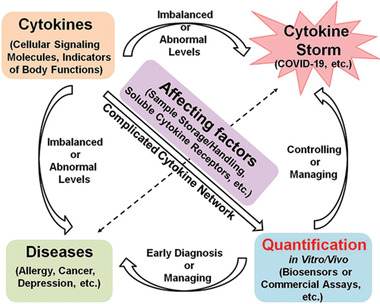
The outline of contents.

This review will introduce cytokines from the perspective of the pathways they trigger and whether they are inflammatory or anti‐inflammatory. The stability of cytokine levels in different body fluids and upon freeze/thawing and sample processing will be discussed. Next, the current biological needs and clinical utility of cytokine detection will be detailed. After that, we summarize recent advances regarding the development of biosensors for cytokine detection both in vitro and in vivo, as well as current commercially available cytokine assays. Their performance (in terms of sensitivity, sample volume, assay time and many other parameters) for cytokines quantifications will be discussed and compared. Finally, we will provide a perspective on the approaches for cytokine detection. The schematics showing the main content of this review is shown in **Figure** **1**. To our knowledge, this is the first comprehensive review on highlighting the biological significance of cytokines and the various methods of their detection although reviews on some related topics were published such as the bioanalytical chemistry of cytokines (2015),^[^ ^41^ ^]^ cytokine immunosensing (2016),^[^ ^19^ ^]^ emerging cytokine biosensors with optical detection modalities and nanomaterial‐enabled signal enhancement (2017),^[^ ^42^ ^]^ and structure‐switching aptamer‐based biosensors for real‐time detection of cytokines (2018).^[^ ^26^ ^]^

## 2 Classification of Cytokines and Their Clinical Significance

Cytokines can be classified into a number of categories including tumor necrosis factors (TNFs), interleukins (ILs), lymphokines, monokines, interferons (IFNs), colony stimulating factors (CSFs), and transforming growth factors (TGFs). Based on their cellular source, cytokines are classified into type 1 cytokines, produced by cluster of differentiation 4 (CD4)+ T‐helper 1 (T_h_1) cells, including IL‐2, IL‐12, IFN‐*γ*, and TNF‐*β*; and type 2 cytokines, produced by CD4+ T_h_2 cells, including IL‐4, IL‐5, IL‐6, IL‐10, and IL‐13.^[^ ^6^ ^]^ Depending on their role cytokines may also be classified as pro‐inflammatory or anti‐inflammatory.^[^ ^6^ ^]^ Pro‐inflammatory cytokines including IL‐1*β*, IL‐6, IL‐8, IL‐12, TNF‐*α*, and interferons among others, facilitate inflammatory reactions and tend to stimulate immunocompetent cells. In contrast, anti‐inflammatory cytokines such as IL‐4, IL‐6, IL‐10, IL‐11, IL‐13, IL‐1 receptor antagonist (IL‐1RA), and TGF‐*β*, inhibit inflammation and suppress immune cells.^[^ ^43^ ^]^ Some cytokines (such as IL‐6) have both pro‐ and anti‐inflammatory properties. These classifications of cytokines, especially the families of pro‐ and anti‐inflammatory cytokines, offer broad perspectives for understanding the pathways triggered by the host response. A single cytokine may be secreted by different cells and have both pro‐inflammatory or anti‐inflammatory activities depending on context, generating multiple immune responses.^[^ ^44^ ^]^ Consequently, a dynamic and ever‐shifting balance between pro‐ and anti‐inflammatory cytokines plays a significant role in the host immune system through mediating and modulating inflammation. Proinflammatory cytokines contribute to the initiation and propagation of autoimmune inflammation, whereas anti‐inflammatory cytokines facilitate the regression of inflammation and recovery from the acute phases of the autoimmune disease.^[^ ^45^ ^]^

This section introduces the pro‐ and anti‐inflammatory cytokines, and their biological and clinical significance, providing a broad and objective understanding about their role in the inflammatory response essential to maintaining our health. **Table** **1** summarizes characteristics of the different cytokines and cell sources; functions of pro‐ and anti‐inflammatory cytokines are also compared.

**Table 1:** Summary of characteristics of different cytokines. Serum samples were taken from 72 healthy subjects including three groups (aged 1–6 years, 7–17 years, and above 18 years). The samples were maintained at 2–8 °C while handling and immediately analyzed utilizing a magnetic bead‐based multiplex immunoassays (Bio‐Plex) (BIO‐RAD Laboratories, Milano, Italy). Cytokine concentrations were measured by Kleiner et al.^[^ ^213^ ^]^ Here cytokine concentrations of the adult group (aged above 18 years) is reported. Plasma samples were taken from ten healthy donors. Samples were processed immediately and measured using a Luminex 100 platform (Luminex, Austin, TX) and BioManager software (Bio‐Rad, Hercules, CA). Cytokine concentrations were tested by Jackman et al.^[^ ^208^ ^]^ Saliva samples were taken from 262 healthy adolescent girls aged 11, 13, 15, 17 years. Samples were assessed annually for three consecutive years. Salivary cytokines were measured using a 96‐well format multiplex electrochemiluminescence immunoassay manufactured by Meso Scale Discovery (MSD, Gaithersburg, MD). Cytokine concentrations were measured by Riis et al.^[^ ^212^ ^]^ Here, saliva concentrations collected in the first year is reported. Tear samples were taken from six female and three male healthy volunteers (age range 25–51). All tear samples were obtained approximately at the same time of the day (16:00–19:00 h) and from the right eye first and then from left eye and were kept cold during collection and stored at −80 °C until assayed. Cytokine levels were determined by multiplex bead analysis in a Luminex IS‐100 instrument (Luminex Corporation, Austin, TX, USA). Cytokine concentrations were measured by Carreno et al.^[^ ^209^ ^]^ Stool samples were taken from healthy adults aged 40–65. These samples were collected in specimen containers and placed into plastic bags, surrounded by frozen gel packs, delivered to the center and stored at −80 °C. Stool cytokines were measured by ELISA. Cytokine concentrations were measured by Vanegas et al.^[^ ^216^ ^]^ Here, cytokine levels collected from participants eating refined‐grain for 2 weeks is reported

					Concentrations [pg mL^−1^] in different in vitro body fluids					
Cytokines	Cytokine type	Cell sources	Half life	Biological functions	Serum	Plasma	Saliva	Tears	Stool	References
IL‐1*β*	Pro‐inflammatory	Monocytes/macrophages	21 min	Principal mediator of the systemic effects of IL‐1; it affects IL‐6‐induced gene expression	–	1.5 ± 1.2	1.5–5.3 × 10^2^	10^2^ ± 2.8	–	^[^ ^207^, ^208^, ^209^, ^210^, ^211^, ^212^ ^]^
IL‐6	Pro‐ and anti‐inflammatory	B and T cells, monocytes, fibroblasts, endothelial cells, and some tumor cells	15.5 h	Inducer of the acute‐phase response as well as specific cellular and humoral immune responses. Inhibition of TNF and IL‐1 production by macrophages	8.5–14	22 ± 8.6	0–27	1.3 × 10^2^ ± 12	0.3 ± 0.1	^[^ ^207^, ^208^, ^209^, ^212^, ^213^, ^214^, ^215^, ^216^ ^]^
IL‐8	Pro‐inflammatory	Monocytes, macrophages, endothelial cells, epithelial cells, hepatocytes, chondrocytes, and tumor cells	24 min	Pro‐inflammatory mediators that orchestrate the recruitment of leukocytes to sites of inflammation	24–36	9.4 ± 3.7	0.4–3.2 × 10^2^	–	–	^[^ ^208^, ^211^, ^212^, ^213^, ^214^ ^]^
IL‐12	Pro‐inflammatory	Phagocytic cells, microglial and dendritic cells	–	Th cell differentiation, TNF‐*α*, IFN‐*γ* synthesis	20–56	1.2 × 10^2^ ± 8.6	0–7.6	47	–	^[^ ^90^, ^208^, ^209^, ^212^, ^213^ ^]^
TNF‐*α*	Pro‐inflammatory	Macrophages, mast cells, NK cells, VSMCs, T‐ and B‐cells	18.2 min	Pro‐inflammatory, neutrophil activation, bone resorption, anticoagulant, tumor necrosis, activate and increase permeability, stimulate adhesion molecules	28–38	5.9 ± 0.4	0–5.8	48 ± 3.3	1.8 ± 0.3	^[^ ^6^, ^91^, ^207^, ^208^, ^209^, ^212^, ^213^, ^216^ ^]^
IFN‐*γ*	Pro‐inflammatory	Macrophages, Th1 cells, Tc cells, B‐cells, Natural killer (NK) cells, VSMCs	–	Pro‐inflammatory, promotes Th1 immune responses/secretion of Th1‐associated cytokines, inhibits ECM synthesis by SMC MHC I expression	(1.2–1.6) × 10^2^	7 ± 2.5	0–7	42 ± 3.6	0.4 ± 0.2	^[^ ^6^, ^208^, ^209^, ^212^, ^213^, ^216^ ^]^
IL‐1RA	Anti‐inflammatory	Monocytes/macrophages, dendritic cells	4–6 h	Inhibition of IL‐1*α*‐ and IL‐1*β*‐mediated cellular activation at the IL‐1 cellular receptor level	(1–1.7) × 10^2^	50 ± 21	–	(3.9 ± 0.9) × 10^3^	–	^[^ ^67^, ^208^, ^209^, ^213^, ^217^ ^]^
IL‐4	Anti‐inflammatory	T cells (Th2), mast cells, B cells, stromal cells	20 min	Promotes Th2 lymphocyte development; inhibition of LPS‐induced proinflammatory cytokine synthesis	6.9–8.1	(2.3 ± 0.5) × 10^2^	–	21 ± 1.6	–	^[^ ^67^, ^207^, ^208^, ^209^, ^213^, ^218^ ^]^
IL‐10	Anti‐inflammatory	Monocytes/macrophages, T cells (Th2), B cells	–	Inhibition of monocyte/macrophage and neutrophil cytokine production and inhibition of Th1‐type lymphocyte responses	8.5–17	38 ± 2.1	0–10	37 ± 0.9	–	^[^ ^67^, ^208^, ^209^, ^212^, ^213^ ^]^
IL‐11	Anti‐inflammatory	Stromal cells, fibroblasts	–	Inhibits proinflammatory cytokine response by monocytes/macrophages and promotes Th2 lymphocyte response	–	–	–	–	–	^[^ ^67^ ^]^
IL‐13	Anti‐inflammatory	T cells (Th2)	–	Shares homology with IL‐4 and shares the IL‐4 receptor; attenuation of monocyte/macrophage function	11–18	(1.1 ± 0.2) × 10^2^	–	47 ± 3.2	–	^[^ ^67^, ^208^, ^209^, ^213^ ^]^

### 2.1 Pro‐Inflammatory Cytokines

The inflammatory response is controlled primarily by cytokines which induce an acute phase response to protect the host against irritation, injury, and infection. This reaction starts with the release of pro‐inflammatory cytokines such as IL‐1*β*, IL‐6, IL‐8, IL‐12, IFN‐*γ*, and TNF‐*α* from the same cell or different cells. The major role of these cytokines is to communicate to surrounding tissues the occurrence of infection or injury. In addition, these cytokines can enter the systemic circulation, producing immune cell activation and significant alterations in host physiology such as fever and the acute‐phase reaction.^[^ ^43^ ^]^

Pro‐inflammatory cytokines have immune properties that can be beneficial to the host against invasion by bacteria and other microorganisms in the immediate environment, or the endogenous flora of the skin and intestinal tract.^[^ ^46^ ^]^ Pro‐inflammatory cytokines released from macrophages are critical in defense against infection.^[^ ^47^ ^]^ Macrophages are the first line of host defense against bacterial infection, playing important roles in the initiation of adaptive immune responses. They are stimulated by bacterial products and release several pro‐inflammatory cytokines including IL‐1, IL‐6, IL‐8, IL‐12, IL‐18, IFN‐*α*/*γ*, and TNF‐*α*. Consistently, these cytokines also directly induce inflammatory activity in macrophages: IL‐1 has direct in vitro cytostatic and cytocidal effects; IL‐6 is considered as a major mediator for immune and inflammatory responses; IL‐12 enhances T‐cell responsiveness; and IFNs mediate host protection against viral infection. These cytokines are related to each other in that they are coordinately released from activated macrophages and modulate the immune response to protect the host.^[^ ^48^ ^]^

It is important to consider that an excessive pro‐inflammatory response may lead to chronic inflammation and disrupt pathways responsible for biological homeostasis causing detrimental health problems such as cancer,^[^ ^49^ ^]^ diabetes,^[^ ^50^ ^]^ cardiovascular diseases,^[^ ^51^ ^]^ gastrointestinal diseases,^[^ ^52^ ^]^ Parkinson's disease, and^[^ ^53^ ^]^ aging and aging‐related diseases.^[^ ^54^ ^]^ Several excellent reviews have comprehensively summarized the essential roles of cytokines in these various medical conditions.^[^ ^3^, ^4^, ^5^, ^51^, ^55^, ^56^, ^57^, ^58^, ^59^ ^]^ Autoimmune diseases^[^ ^60^, ^61^ ^]^ (such as type 1 diabetes, rheumatoid arthritis, inflammatory bowel disease and multiple sclerosis) are conditions in which the immune system attacks the self mistakenly. Contributions of individual cytokines and chemokines to multiple autoimmune diseases are discussed by Santamaria.^[^ ^62^ ^]^ Pro‐inflammatory interferons play essential roles in the development of autoimmune diseases. There are reports on the role of IFN‐*γ* in the pathogenesis of autoimmune disease and its impact on associated co‐morbidities and side effects of therapeutic interventions in the absence or presence of cancer.^[^ ^63^, ^64^ ^]^ In this regard, in a pre‐clinical mouse model of autoimmunity, chronic IFN‐*γ* expression has been shown and the mice gradually develop mild to moderate active IFN‐*γ*‐driven autoimmune disease.^[^ ^63^ ^]^ Such models allow the study of inflammation and autoimmune disease progression under different threshold levels of IFN‐*γ* protein that, when crossed, leads to much stronger immunopathology. Recently, Bae et al. reported that pathway‐based integration of multi‐omics data can provide systemic and cellular insights about how chronic inflammation driven by IFN‐*γ* results in the development of autoimmune diseases with specific etiopathological features.^[^ ^65^ ^]^

Research has shown that during the growth and spread of tumors, pro‐inflammatory cytokines such as IL‐1, IFN‐*γ*, and TNF‐*α* induce chemokines that attract neutrophils which are key factors in the generation of reactive oxygen species and carcinogenesis.^[^ ^46^ ^]^ Relevant to such research, elevated levels of pro‐inflammatory cytokines (IL‐6, IL‐1*β*, and TNF‐*α*) are observed in mouse models of Parkinson's disease.^[^ ^66^ ^]^ Additionally, pro‐inflammatory cytokines induce adhesion molecules and metalloproteinases which permit specific mechanisms for tumor invasion. As a whole, such excessive pro‐inflammatory responses need to be regulated and controlled or else they may result in pathological states related to the aberrant expression of immune mediators.

### 2.2 Anti‐Inflammatory Cytokines

The anti‐inflammatory cytokines such as the IL‐1 receptor antagonist, IL‐4, IL‐6, IL‐10, IL‐11, IL‐13, and TGF‐*β* are a series of immunoregulatory molecules which inhibit the excess inflammatory response of pro‐inflammatory cytokines.^[^ ^67^ ^]^ For instance, IL‐10 is a potent anti‐inflammatory cytokine with immunoregulatory functions that inhibit the production of several pro‐inflammatory cytokines. IL‐10 also has an anti‐inflammatory effect on eosinophils, basophils, and mast cells, and thus plays a major role in the control and regulation of allergy and asthma.^[^ ^68^ ^]^ The physiologic properties of anti‐inflammatory cytokines have been recognized.^[^ ^67^ ^]^

Under physiologic conditions, these cytokines limit the potentially injurious effects of sustained or excess expression of pro‐inflammatory reactions. These anti‐inflammatory cytokines have already proven beneficial under various clinical conditions associated with excess inflammation. For example, anti‐inflammatory cytokines can be used as drugs to treat inflammation‐related diseases. However, cytokine therapy also suffers from a number of limitations as compared to anti‐inflammatory biologics such as neutralizing antibodies.^[^ ^69^ ^]^ For example, specific anti‐inflammatory cytokines might effectively inhibit arthritis by affecting innate immune cells or interfering with the activation of B cells or T cells.^[^ ^70^ ^]^ IL‐35 is an anti‐inflammatory cytokine that regulates T cell function and suppresses pathogenic cells such as T_h_1 and T_h_17 cells, and thus ameliorates the severity of collagen‐induced arthritis.^[^ ^57^ ^]^ In contrast, under pathologic conditions these anti‐inflammatory mediators may overcompensate and suppress the immune response, exposing the host to systemic infection.^[^ ^67^ ^]^ Although research suggests that endogenous IL‐10 has protective effects in severe sepsis by reducing the production of TNF, the overproduction of IL‐10 resulting in excessive TNF downregulation might be deleterious due to the impairment of the antibacterial activity provided by TNF.^[^ ^71^ ^]^

### 2.3 Biological Consequences of Imbalanced Cytokines in a Clinical Context

Considering innate and adaptive immunity, both pro‐ and anti‐inflammatory cytokines have major biological and clinical significance on immune cell differentiation, inflammation, angiogenesis, tumorigenesis, neurobiology, viral pathogenesis, atherosclerosis, cancer, and aging.^[^ ^72^ ^]^ **Table** **2** illustrates different typical diseases related to the interactions of various cytokines. This supports the model that cytokines act as biomarkers for a variety of autoimmune and inflammatory diseases.

**Table 2:** Multiple cytokines related to different biological conditions

Diseases	Relate cytokines	References
Autoimmune diseases	IL‐1, IL‐2, IL‐6, IL‐12, IL‐15, IL‐16, IL‐17, IL‐18, IL‐23,TNF‐*α*, IFN‐*α*, IFN‐*γ*	^[^ ^62^ ^]^
Allergy	IL‐1, IL‐4, IL‐5, IL‐9, IL‐10, IL‐13	^[^ ^219^ ^]^
Alzheimer's disease	TNF‐*α*, TGF‐*β*, IL‐1, IL‐4, IL‐6, IL‐10	^[^ ^220^ ^]^
Atherosclerosis	TNF‐*α*, IFN‐*γ*, TGF‐*β*, IL‐1, IL‐2, IL‐4, IL‐5, IL‐6, IL‐8, IL‐10, IL‐12, IL‐17, IL‐18, IL‐20, IL‐33, IL‐37	^[^ ^221^ ^]^
Cardiovascular disorders	TNF‐*α*, TGF‐*β*, IL‐1, IL‐6, IL‐10, IL‐17, IL‐18	^[^ ^51^ ^]^
Cancer	TNF‐*α*, TRAIL, IL‐6, IL‐10, IL‐12, IL‐17, IL‐23	^[^ ^3^ ^]^
Depression	TNF‐*α*, IFN‐*γ*, IL‐1, IL‐2, IL‐6	^[^ ^222^ ^]^
Gastrointestinal diseases	TNF‐*α*, IFN‐*γ*, TGF‐*β*, IL‐1, IL‐4, IL‐6, IL‐8, IL‐10	^[^ ^223^ ^]^
Sepsis	TNF‐*α*, IFN‐*γ*, TGF‐*β*, MIF, IL‐1, IL‐6, IL‐4, IL‐10, IL‐12	^[^ ^224^ ^]^
Aging	IL‐6, IL‐8, IL‐10, IL‐13, TNF‐*α*, IFN‐*γ*	^[^ ^225^ ^]^

Our immune system acts as a “double edged sword” that can either heal or harm that is based on differentiating between the “self”’ and the “non‐self”’ and destroying only those tissues that are recognized as “non‐self.” Failure of immune recognition of the body's normal constituents as “self” results in inflammation and tissue damage. Inflammation is a complex biological response of the body to injury and infection which is regulated and mediated by the balance of inflammatory activities associated with cytokines. The imbalance between tissue homeostasis and inflammatory cytokines, and the unregulated pro‐ and anti‐inflammatory cytokine levels, can lead to significant negative health impacts.^[^ ^73^ ^]^ For example, in the pathogenesis of inflammatory bowel disease,^[^ ^5^ ^]^ risk factors such as microorganisms, infections and cytokines may initiate alterations in epithelial barrier function thereby allowing the translocation of luminal antigens (for example, bacterial antigens from the commensal microbiota) into the bowel wall. Subsequently, excessive cytokine responses to such environmental triggers may cause subclinical or acute mucosal inflammation in a susceptible host.^[^ ^74^ ^]^ To suppress this inflammation, the administration of recombinant anti‐inflammatory cytokines or the neutralization of pro‐inflammatory cytokines could be used for both the prevention and the therapy of chronic intestinal inflammation.^[^ ^5^, ^74^, ^75^, ^76^ ^]^ As a topical example, with the spread COVID‐19 pandemic, research has found that there is high disparity in the susceptibility of COVID‐19 severity in individuals. To identify the underlying factors for this disparity, Gou et al.^[^ ^77^ ^]^ developed a proteomic risk score (PRS) based on 20 blood proteomic biomarkers which predicts the progression to severe COVID‐19. The authors discovered that the PRS is positively associated with pro‐inflammatory cytokines mainly among the elderly, but not younger individuals, suggesting that profiling cytokines in the gut may underlie the predisposition of normal individuals to severe COVID‐19.^[^ ^78^ ^]^ A discussion on the possible systemic production and injection of cytokines in the gut of COVID‐19 patients can be found a recent perspective.^[^ ^79^ ^]^

**Figure 2:**
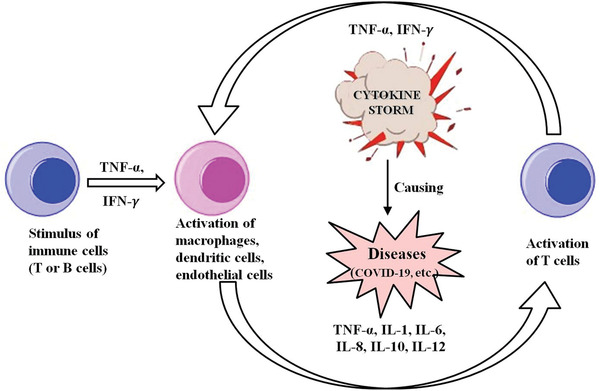
Proposed mechanism of cytokine release syndrome.

Additionally, the excessive or uncontrolled release of proinflammatory cytokines may contribute to the potentially life‐threatening cytokine release syndrome (CRS), a condition with an immune system gone awry and an inflammatory response out of control.^[^ ^80^ ^]^ CRS can be triggered by many factors including infections, administration of natural and bispecific antibody pharmaceuticals and following adoptive T‐cell therapies for cancer. CRS presents with a variety of symptoms ranging from mild, flu‐like symptoms to severe life‐threatening manifestations of the overactive inflammatory response.^[^ ^81^ ^]^ **Figure** **2** illustrates a patho‐mechanism whereby activation of T‐cells or lysis of immune cells induces the production of IFN‐*γ* or TNF‐*α*. In turn, this can activate macrophages, dendritic cells and other immune cells. These cells then further release several proinflammatory cytokines such as IL‐6, IL‐10, IL‐2, and IL‐8, contributing to a positive feedback loop to activate T‐cells that are capable of causing life‐threatening toxicities. Accumulating evidence suggests that severe cases of COVID‐19 presenting with high viral loads, respiratory distress, and pulmonary damage might relate to surges in cytokines levels due to CRS.^[^ ^8^ ^]^ Initial research has suggested that elevated serum IL‐6 levels are associated with respiratory failure and adverse clinical outcomes in COVID‐19.^[^ ^82^, ^83^ ^]^ Further studies have demonstrated persistently raised levels of the additional cytokines such as TNF‐*α* and IL‐1RA in severe cases.^[^ ^84^, ^85^ ^]^ Yang et al.^[^ ^84^ ^]^ examined 48 cytokines in the plasma samples from 53 COVID‐19 cases and found that 14 cytokines were significantly elevated. Serial detection of IP‐10, MCP‐3, and IL‐1RA in 14 severe cases showed that a continuous high level of these cytokines is associated with disease deterioration and fatal outcomes. Given these findings, immunosuppression using tocilizumab to reverse CRS and consequently lowering mortality has entered clinical trials to treat COVID‐19.^[^ ^8^ ^]^ Thus, the evaluation of the rise in cytokines levels is essential to diagnose and manage the complications of CRS in clinical immunotherapies^[^ ^86^, ^87^ ^]^

## 3 Factors Affecting Cytokine Quantification in Biological Fluids

Many factors can affect the measurement of cytokines in biological fluids as discussed by Heney and Whicher in 1990s,^[^ ^88^ ^]^ such as 1) the quality of the cytokines assays, 2) the nature of cytokines under biological conditions affected by cytokine‐binding proteins, inhibitors and soluble cytokine receptors, 3) interferences in the matrix of biological samples causing false positive signals, and 4) assay standardization, which is a popular and bottleneck problem for the majority protein assays. Additionally, the handling of biological samples also has a remarkable impact on cytokines detection due to the fact that cytokine measurements normally involve the process by which samples are handled from the point of sampling to the laboratory.^[^ ^89^ ^]^ Furthermore, the short half‐life of cytokines (Table 1), their binding to soluble receptors as well as the production or the potential degradation of cytokines affects the precision of cytokine measurement, analyses and interpretations.^[^ ^90^ ^]^ For example, the half‐life of TNF‐*α* is 18.2 min.^[^ ^91^ ^]^ Therefore, to make measurement accurate, conditions related to sample collection and handling should be reported with the quantification data. In this section we specifically discuss the stability of cytokines in body fluids such as plasma and serum, the influence of sample handing conditions and freeze‐thaw cycles on cytokines levels, and the effects of soluble cytokines receptors, all of which may impact the reliability of cytokines measurements.

### 3.1 Effects of Blood Sample Processing on Cytokine Stability

Cytokines are measured in different body fluids including blood, saliva, tears, urine, and stool. Blood is close to the internal environment of an individual, reflecting the state of individual cells, tissues, organs and the body as a whole. Analysis of cytokine levels in clinical blood specimens, especially plasma or serum is important for disease diagnosis as the subtle change in levels may reflect the status of immune function.^[^ ^92^ ^]^ However, the majority of cytokines are known to have a short half‐life (Table 1) in vivo and are subject to rapid degradation during sample collection and preparation. This results in false negative signals if appropriate blood handling procedures are not adopted.^[^ ^93^ ^]^

Serum and plasma are derived from whole blood and handled by differently after blood collection. Serum is the soluble part of clotted blood and is obtained following blood coagulation. Blood cells may be activated during this clot formation and cytokines may be released from platelets into the serum as a result (such as IL‐1, IL‐6, and IL‐8).^[^ ^4^ ^]^ Plasma represents the soluble fraction of anticoagulated blood.^[^ ^4^ ^]^ Prior to plasma separation from whole blood, leukocytes can secrete cytokines in vitro and change cytokines levels in plasma.^[^ ^94^ ^]^ To obtain plasma, various anticoagulants can be used before the removal of blood cells, such as ethylenediaminetetraacetic acid (EDTA) and lithium/sodium heparin, thereby inhibiting both coagulation and the activation of the complement system. Studies have suggested that the use of various anticoagulants, endotoxins tube contamination, and delays in blood processing (centrifugation) can have a major impact on cytokines concentrations in plasma or serum and can result in falsely increased or decreased cytokine measurements.^[^ ^95^ ^]^ For instance, heparin, an anticoagulant in whole blood processing, can induce cytokines release from monocytes. Lithium heparin and sodium citrate were shown to affect levels of IL‐6 and TNF‐*α*,^[^ ^96^ ^]^ which could be attributed to anticoagulant‐induced release of cytokines by blood cells, notably in heparin plasma but not in EDTA plasma. Friebe and Volk reported the stability of TNF‐*α*, IL‐6, and IL‐8 in blood samples and found that levels of TNF‐*α* and IL‐8 increase in heparin plasma and serum, but their concentrations were stable in EDTA plasma.^[^ ^97^ ^]^ In contrast, IL‐6 levels were stable for 8 h in all blood types. The higher cytokine levels in serum compared with those in plasma suggest that the coagulation process promotes cytokine release. This result is consistent with those of previous studies.^[^ ^98^, ^99^ ^]^ Plasma collection with the use of EDTA seems to bring the most consistent results and more closely resembles data obtained in serum. In summary, EDTA plasma seems to be the most suitable for cytokine measurements, primarily for stability reasons.^[^ ^100^ ^]^ Additionally, quick sample preparation is usually recommended, although there is always a time gap between blood collection and arrival in the laboratory for testing.

### 3.2 Effects of Sample Storage on Cytokine Stability

To obtain reliable results, many studies have examined the effects of storage on cytokine levels in blood. Cohen et al.^[^ ^94^ ^]^ evaluated the impact of sample storage on IL‐6, IL‐10, IFN‐*γ*, and IL‐2 measurements in plasma. Their results have shown that whole blood storage at room temperature results in decreased cytokines levels but that whole blood storage at 4 °C results in cytokines stability. A recent study by Vincent et al. evaluated the effect on cytokine stability of storage duration prior to freezing of serum, and compared the results to plasma samples obtained from patients with systemic lupus erythematosus (SLE).^[^ ^101^ ^]^ In this study, patients’ serum and plasma samples were prospectively stored at 4 °C for pre‐determined periods between 0 and 30 days, prior to freezing. Almost all analyzed cytokines (11 out of 12) were stable when stored for up to 30 days at 4 °C prior to freezing. Only a single analyte, chemokine (C‐C motif) ligand 19 (CCL19) showed significant signal degradation from the fourth day of storage at 4 °C. Cytokines levels were more stable in unseparated serum compared to plasma for most analytes with the exception of IL‐37 which appeared slightly more stable in plasma. This study suggests a maximum 3 days of storage at 4 °C for unseparated serum samples. Recently Valaperti et al. analyzed the variability of cytokine levels over time in whole blood before and after cell separation to establish a protocol that reflects the best storage conditions for reliable measurements.^[^ ^102^ ^]^ This research demonstrated that many cytokines are stable for a brief time after sample collection at room temperature. It is recommended that freshly collected whole blood samples be quickly processed and frozen to avoid false positive results, a finding that is further supported by Panicker et al. who studied the effect of snap‐freezing and refrigeration at the time of collection from cervical mucous.^[^ ^93^ ^]^ TNF‐*α*, IFN‐*γ* and IL‐1*β*, were significantly different between the pairs with refrigerated samples showing higher levels for each of these cytokines. This finding suggests that refrigeration of mucous samples immediately after collection would allow for better conservation of the cytokines in cervical mucous.

### 3.3 Effect of Freeze‐Thaw on Cytokine Stability

A review by Simpson et al. summarized the stability of 33 cytokines when samples were stored at various temperatures or exposed to repetitive freeze‐thaw cycles.^[^ ^103^ ^]^ Assessment of freeze‐thaw stability is an important consideration for the measurements of cytokines because of the common use of previously thawed samples. The levels of cytokines can either be stable, increase or decrease after multiple freeze–thawing cycles, and is different for each cytokine.^[^ ^104^ ^]^ In general, most cytokines are stable for up to three freeze–thaw cycles.^[^ ^90^ ^]^ Jae et al. assessed the impact of repeated freezing and thawing on plasma and serum concentrations of different cytokines. They found that the levels of IFN‐*γ* and IL‐8 were stable in both plasma and serum during repeated freeze‐thaw cycles.^[^ ^105^ ^]^ However, concentrations of certain cytokines change with each successive freeze–thaw cycle becoming significant after three cycles.^[^ ^90^ ^]^ Henno et al. studied the effect of freezing and thawing on cytokines stability in EDTA and citrate plasma and reported that there were no significant change in the cytokine levels in plasma frozen and thawed up to three times.^[^ ^106^ ^]^ However, after freezing and thawing six times, there was a slight but biologically significant decrease in the IL‐1*β* level and an increase in the CCL5 level in EDTA plasma. This suggests a maximum of three freeze‐thaw cycles for sample handling in order to perform the accurate cytokines analysis.

On a final note, there are a wide variety of reported cytokines storage and freeze‐thaw stability studies. IL‐6 and TNF‐*α* are the most widely studied cytokines in regard to temperature stability. For a few cytokines, a clear consensus can be reached as to storage safety at particular temperatures, but in most, more research needs to be undertaken and we advise clinicians and researchers to use caution in interpreting cytokines concentrations after a long period of storage or several freeze‐thaw cycles. In general, in order to maintain stable cytokines levels for accurate measurements, samples should undergo minimal freeze–thawing.

### 3.4 Antagonistic and Agonistic Effects of Soluble Cytokine Receptors on Cytokine Detection

Soluble cytokine receptors or cytokine binding proteins (e.g., IL‐18 bp) arise from the proteolytic cleavage of membrane‐bound receptors or from the translation of alternatively spliced mRNAs which are released from the cells and appear in biological fluids or tissue culture supernatants.^[^ ^107^ ^]^ These receptors, acting as competitive inhibitors, have antagonistic effects on their respective cytokines in vitro. There are many examples illustrating that most soluble cytokine receptors can interfere and compete with cell surface receptors for the binding of free cytokines. Consequently, cytokine receptors prevent cytokines from binding their specific membrane receptors and generating a signal, leading to inhibition of cytokines activity.^[^ ^107^ ^]^ The antagonistic effects of soluble cytokine receptors may play an important role in the down‐regulation of immune responses and in the inhibition of “overactivity” of some cytokines. For example, Levine reported that soluble IL‐1 receptors can attenuate excessive IL‐1 bioactivity by preferentially binding IL‐1*β*.^[^ ^108^ ^]^

Despite the fact that most soluble cytokine receptors have the ability to function as competitive inhibitors of cytokines, several receptors may potentiate the activity of their own cytokines in vivo or have properties that are consistent with an added role as carrier proteins. This type of soluble receptor enhances, rather than inhibits, the activity of cytokines by interacting with their signal transducing subunit, thus generating a signal (i.e., the soluble IL‐6 receptors (sIL‐6R) and glycoprotein 130 (gp130)). In contrast to the antagonistic effect of soluble IL‐1 receptors on IL‐1 signals, Levine reported the agonistic effects of sIL‐6R for the amplification of IL‐6 signals.^[^ ^108^ ^]^ Therefore, binding of a cytokine by its soluble receptor may improve the molecular stability of the cytokine leading to reduced activity. This hypothesis is consistent with the idea that the binding of the bioactive TNF trimer to soluble TNF receptors slows its breakdown into inactive monomers resulting in increased biological activity after long‐term incubation.^[^ ^107^ ^]^

These antagonistic and agonistic effects of soluble cytokine receptors can potentially affect the detection of cytokines. A study^[^ ^109^ ^]^ shows that in some cases, like inflammatory diseases, the presence of soluble cytokine receptors in biological fluids may interfere with immunoassays such as bead‐based multiplex immunoassays and ELISA. Several cytokines, notably IL‐1*β*, TNF‐*α*, and IL‐6, may bind to soluble receptors generating bound forms which may not be recognized by immunoassays. For example, in cancer patients, competitive immunoassays are often detectable for TNF‐*α*, but ELISA assays detect no TNF‐*α* in plasma of cancer patients, in agreement with the bioassay data.^[^ ^88^ ^]^ Engelberts et al.^[^ ^110^ ^]^ studied these effects and showed that TNF‐*α* bound to the p55 TNF receptor was not well recognized by sandwich ELISA assays. In addition, in the case of IL‐6, plasma contains several bound forms of IL‐6 with molecular weights ranging from 50–150 to 400–500 kDa, which react poorly with some antisera, and consists of complexes with the soluble form of the IL‐6 receptor. This has led to controversy over what concentrations of IL‐6 are actually present in plasma. Most immunoassays find that concentrations of IL‐6 in normal plasma are undetectable or range between 10 and 75 ng L^−1^, with levels rising to 1–2 µg L^−1^ in sepsis or, exceptionally to 200 µg L^−1^ in meningococcal disease. However, May et al.^[^ ^111^ ^]^ have reported that most assays recognize only IL‐6 of low molecular mass. Using a monoclonal antibody that recognizes the high molecular mass forms, they have shown concentrations of IL‐6 in normal plasma of 1–10 µg L^−1^, and, in a serum sample from a patient after bone marrow transplantation, a concentration of 5–10 mg L^−1^. Therefore, it is necessary to know exactly which component of a cytokine or cytokine complex that an assay is measuring, and ideally levels of soluble receptors should be taken into account.

## 4 Quantification of Cytokines

### 4.1 Detection of Cytokines In Vitro and In Vivo

Cytokines, considered as biomarkers for many diseases plays an important role for the assessment of physiological and pathological processes. Quantifying cytokines can provide highly valuable clinical information to measure the immune status of the host and to adjust therapies in different inflammatory diseases such as sepsis and cancer.^[^ ^24^ ^]^ Cytokines are present in different in vitro body fluids (blood, tears, urine, and stool) and in vivo body fluids (interstitial fluids, cerebrospinal fluids, and gut), and can be detected in vitro or in vivo.

**Figure 3:**
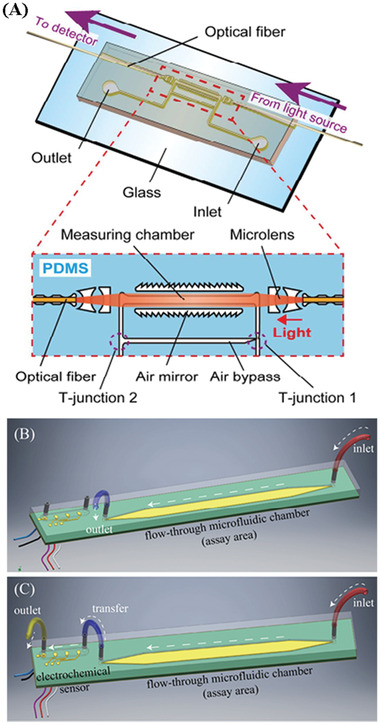
Schematic illustration of A) The PhLoC with integrated optical and microfluidic components. Reproduced with permission.^[^ ^112^ ^]^ Copyright 2016, American Chemical Society. B) A 3D graphical representation of the unit cell fluidic arrangement during the initial phase of the assay. C) A 3D graphical representation of the unit cell fluidic arrangement during the second phase of the assay, detailing fluidic ports and connections between assay area and electrochemical sensor test cartridge of a Proxim handheld instrument. Reproduced with permission.^[^ ^117^ ^]^ Copyright 2018, MDPI.

Cytokine detection in vitro is flexible and effective, and has been utilized widely across the research community. A vast range of samples including cells, tissues, and body fluids have been used for in vitro cytokines tests. There are multiple techniques which are being used for cytokines measurements in vitro, including ELISA, PCR, and advanced biosensors including POC testing. However, there are some challenges to in vitro cytokines detection. One important issue is that they require accurate and consistent processing of samples to avoid changes of cytokine concentrations before testing. Another challenge is to realize real‐time detection for in vitro analysis. Not only must the analytical requirements such as high sensitivity and precision be met, but the test must be fast, and integrated for ease‐of‐use and in real time. To deal with these challenges, LoC devices have been developed to realize fast and real‐time cytokine detection in vitro.^[^ ^112^, ^113^, ^114^, ^115^ ^]^ For example, Usuba et al. fabricated a photonic lab‐on‐a‐chip (PhLoC) with a microfluidic structure for rapid IL‐2 detection.^[^ ^112^ ^]^ The PhLoC is shown in **Figure** **3**A, including optical components, the measuring chamber, the air bypass, and other flow channels for the introduction and flushing of solutions. In their work, the flow channel only enabled the introduction of solutions into the measuring chamber and improved the immobilization of antibodies on the surfaces of the measuring chamber. The IL‐2 secreted from lymphocytes could be measured within 15 min for concentrations ranging from 50 to 10^3^ pg mL^−1^. In the lab‐on‐a‐chip devices, microfluidics provides an effective solution for achieving more rapid and efficient in vitro detection, because 1) microfluidic channels have large surface‐to‐volume ratios, accelerating antigen‐antibody reactions, 2) the microfluidic platform minimizes the consumption of expensive reagents and precious samples, and 3) multiplexed analyses can be implemented by integrating multiple sensors into channels. Consequently, in the past decade, microfluidic techniques have been widely developed for quantitative measurements of secreted cytokines. Cui et al. reported a highly integrated microfluidic device that allows for on‐chip isolation, culture, and stimulation, as well as sensitive and dynamic cytokine profiling i immune cells.^[^ ^113^ ^]^ This microfluidic sensing chip was integrated with cytometric fluorescent microbeads for real‐time and multiplexed monitoring of cytokine secretion dynamics required a relatively small extracted sample volume (160 nL) and a short assay time of less than 30 min. Such automated, rapid, and high‐throughput microfluidics‐based optical biosensing platforms can potentially help unleash the mechanisms of systemic immune responses and enable efficient assessments of the pathologic immune status. Recently, Liu et al. developed a microfluidic chip based aptasensor for electrochemical detection of IFN‐*γ* in human serum with a linear range of 10–500 pg mL^−1^ and the lowest detection limit of 6 pg mL^−1^.^[^ ^31^ ^]^ Due to ease of use, low cost and rapid diagnosis of disease, POC assays also play important roles for cytokine detection in vitro. For example, a POC assay was developed for real‐time monitoring and management of IL‐6 release syndrome and sepsis.^[^ ^116^ ^]^ This device demonstrated good sensitivity (2.0 pg mL^−1^) and a wide dynamic range (from 2.0 pg mL^−1^ to 15 ng mL^−1^) that could be implemented for on‐site evaluation with results available as quickly as 15 min, with enhanced diagnostic speed and accuracy. Evans et al.^[^ ^117^ ^]^ developed a novel POC biosensor system on a printed circuit board (PCB) for IFN‐*γ* detection. This full in‐line assay system consists of an assay area and an electrochemical cell at the surface of a PCB as shown in Figure 3B,C. It was demonstrated that the entire assay could be completed within 8 min, which significantly reduced the test time comparing with conventional ELISA.

In vivo, cytokines have complex networks to regulate immune and inflammatory responses. Many studies have focused on detecting cytokines in vitro to understand how cytokines are trafficked and how their expression is regulated. However, inappropriate processing and storage conditions of samples may influence cytokines levels, causing inaccurate measurements. Additionally, the non‐homogeneous distribution of cytokines also makes in vivo localized cytokine detection essential to understand cytokine expression and release dynamics. Thus, there is high demand to capture and quantify in vivo cytokines in body fluids (blood, interstitial fluids, cerebrospinal fluids, gut fluids, and tears) in real time. After the successful demonstration of an electrochemical immunosensor for detection of IL‐6 in vivo,^[^ ^30^ ^]^ Qi et al. pioneered the development of an optical fiber based immunosensing device for spatially localized cytokine detection in discrete brain regions with a sensitivity of 3.9 pg mL^−1^.^[^ ^118^ ^]^ An increase in fluorescence detection of spatially localized intrahippocampal IL‐1*β* release was observed following a peripheral lipopolysaccharide challenge in Sprague–Dawley rats. This novel immunosensing technology represented an opportunity for unlocking the function of neuroimmune signaling. Recently this in vivo device was successfully used for investigating IL‐1*β* extracellular release in the dorsal hippocampus after an acute stressor induced by exposing male Sprague–Dawley rats to inescapable tail‐shock.^[^ ^119^ ^]^

Considering the potential complexity of optical fibers for in vivo measurements, further steps have been taken to replace the optical fiber with transducers based on stainless steel (SS) wires. With the guidance of an implanted intrathecal catheter, this SS based sensing device could be inserted along the spinal cord of rats to quantify in vivo intrathecal IL‐1*β* concentrations permitting monitoring of the molecular signals of neuropathic pain.^[^ ^120^ ^]^ This in vivo cytokine assay established a possible correlation between biochemical spinal marker expression and in vivo quantification of IL‐1*β*. Although these deployable sandwiched based immunosensors were able to capture and measure cytokines in vivo, they belong to two‐step assays (in vivo capture and subsequent in vitro quantification) resulting in an overall process delay. Immune reactions associated with cytokines as we know are often extremely dynamic and may be transient in nature. Thus, access to single step real‐time detection of cytokines in vivo can provide more accurate and reliable information. In this regard, structure‐switching molecules have demonstrated their potential in real‐time detection of analytes.^[^ ^26^ ^]^ A molecular beacon aptamer based biosensing device has been developed toward the near real‐time^[^ ^36^ ^]^ and real‐time monitoring of IFN‐*γ*.^[^ ^121^ ^]^ Such a platform has been proven sensitive for the detection of cytokines in the pg mL^−1^ range. Specifically, we developed a proof‐of‐concept in vivo sensing device for simultaneously monitoring IFN‐*γ* at a sensitivity of 10 pg mL^−1^ and the subsequent release of aspirin triggered by IFN‐*γ*. This in vivo cytokine assay based on the aspirin intercalating hairpin aptamer realized continuous monitoring of IFN‐*γ*. This technology thus provides a promising strategy for in vivo real‐time monitoring of cytokines and subsequent drug delivery toward precise theranostics. However, background signal drifting and stability under in vivo conditions are potential challenges associated with structure‐switching aptamer‐based in vivo cytokine sensing. Ratiometric detection^[^ ^122^ ^]^ and aptamer modification^[^ ^123^ ^]^ are promising solutions for these challenges.

### 4.2 Biosensors for Cytokines Detection

**Figure 4:**
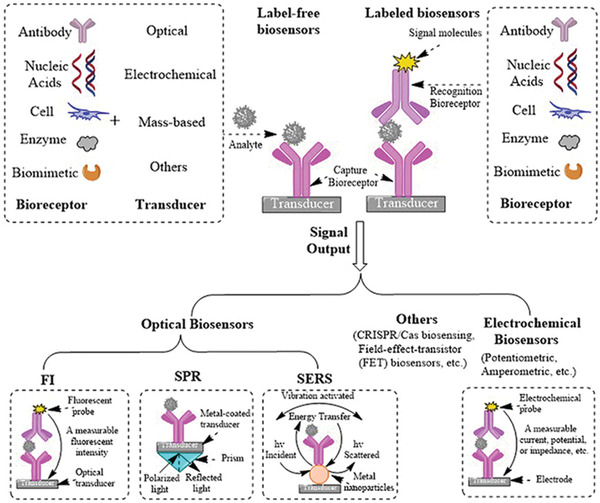
Schematic illustration of label‐free and labeled biosensors (FI, SPR, SERS, and EC) using antibodies.

The ultralow concentration of cytokines (generally in the pm range), and extremely dynamic, transient cytokine secretion processes make cytokines quantification challenging. By integrating with nanotechnology, biosensors as the analytical devices for the detection of analytes that combines biological components with a physicochemical detector, has demonstrated great potential for sensing. Such cytokines biosensors^[^ ^124^ ^]^ can rely on fluorescence or electrochemical signal readouts to quantify intracellular and extracellular cytokines in non‐real‐time^[^ ^39^, ^125^ ^]^ or in real‐time.^[^ ^31^, ^126^ ^]^ They can also use other transducing elements. **Table** **3** compares various biosensors for cytokine detection based on different signaling strategies such as fluorescence immunoassays (FI), surface plasmon resonance detection (SPR), electrochemical‐based methods (EC), surface enhanced Raman spectroscopy (SERS), colorimetric, CRISPR/Cas signal amplification linked immunosorbent assay (CLISA) and other methods, in terms of their performance (i.e., sensitivity, and levels of samples required). To understand the scheme of the above biosensors we mentioned, here we take label‐free and labeled biosensors using antibodies as examples, showing the basic scheme and signal readouts of these biosensors in **Figure** **4**, and more detailed information of each biosensor for cytokine detection is introduced respectively in the following part. This section will introduce recent advances of the different strategies used for the in vitro or in vivo detection of cytokines and their analytical performance will be compared and discussed.

**Table 3:** Overview of biosensors for cytokine detection based on different detection techniques

Cytokines	Detection technique	Detection limit	Linear range	Sample volume	Assay time	Reference
IL‐2, IL‐4, IL‐6, IL‐10, IFN‐*γ*, TNF‐*α*	FI	41 pg mL^−1^	41–10^4^ pg mL^−1^	50 µL	≈3 h	^[^ ^226^ ^]^
TNF‐*α*	FI	20 pg mL^−1^	10^5^–10^6^ pg mL^−1^	–	Near real time	^[^ ^227^ ^]^
IFN‐*γ*	FI	1.5 × 10^4^ pg mL^−1^	(0.2–8) × 10^5^ pg mL^−1^	–	≈6 h	^[^ ^130^ ^]^
IFN‐*γ*	FI	2 pg mL^−1^	5–10^2^ pg mL^−1^	–	≈30 min	^[^ ^33^ ^]^
IFN‐*γ*	FI	0.1 pg mL^−1^	0.1–1.5 × 10^3^ pg mL^−1^	–	≈2 h	^[^ ^131^ ^]^
IFN‐*γ*	FI	2 pg mL^−1^	0–10^2^ pg mL^−1^	–	≈45 min	^[^ ^228^ ^]^
IFN‐*γ*, TNF‐*α*	FI	21 pg mL^−1^	0–3.6 × 10^2^ pg mL^−1^	–	≈40 min	^[^ ^128^ ^]^
IL‐1*β*	FI	3.2 pg mL^−1^	3.5–2 × 10^2^ pg mL^−1^	5–10 µL	–	^[^ ^35^ ^]^
IL‐20	FI	0.2 pg mL^−1^	2–2 × 10^4^ pg mL^−1^	5 µL	–	^[^ ^229^ ^]^
IL‐1*β*	FI	4.7 pg mL^−1^	13–2 × 10^2^ pg mL^−1^	1 µL	–	^[^ ^230^ ^]^
IL‐1*β*	FI	10 pg mL^−1^	25–4 × 10^2^ pg mL^−1^	–	–	^[^ ^125^ ^]^
IL‐6	FI	1 pg mL^−1^	1–4 × 10^2^ pg mL^−1^	1 µL	–	^[^ ^37^ ^]^
IL‐6	FI	0.1 pg mL^−1^	0.4–4 × 10^2^ pg mL^−1^	1 µL	–	^[^ ^231^ ^]^
IFN‐*γ*	FI	10^3^ pg mL^−1^	5 × 10^3^‐1 × 10^5^ pg mL^−1^	–	Near real time	^[^ ^32^ ^]^
IL‐2, IL‐4, IL‐6	SPR	5–20 pg mL^−1^	10–10^4^ pg mL^−1^	1 µL	≈40 min	^[^ ^24^ ^]^
IL‐6	SPR	10 pg mL^−1^	10–10^2^ pg mL^−1^	–	≈30 min	^[^ ^232^ ^]^
IL‐6, TNF‐*α*	SPR	5 pg mL^−1^	4–5 × 10^2^ pg mL^−1^	–	–	^[^ ^137^ ^]^
IL‐6, IL‐4, IL‐10,TNF‐*α*	SPR	20 pg mL^−1^	10–10^4^ pg mL^−1^	1 µL	≈30 min	^[^ ^233^ ^]^
IFN‐*γ*	SPR	5 × 10^4^ pg mL^−1^	(0.5–8) × 10^5^ pg mL^−1^	800 µL	Real time	^[^ ^136^ ^]^
IL‐6	SPR	10^4^ pg mL^−1^	10^4^–2 × 10^5^ pg mL^−1^	100 µL	Real time	^[^ ^138^ ^]^
TGF‐*β*1	EC	10 pg mL^−1^	15–3 × 10^3^ pg mL^−1^	25 µL	≈60 min	^[^ ^234^ ^]^
IL‐6, IL‐1*β*, TNF‐*α*	EC	5 pg mL^−1^	5–2 × 10^2^ pg mL^−1^	–	–	^[^ ^28^ ^]^
IFN‐*γ*	EC	1.6 pg mL^−1^	2.5–2 × 10^3^ pg mL^−1^	5 µL	≈200 s	^[^ ^152^ ^]^
IFN‐*γ*	EC	0.2 ng mL^−1^	0.2–2.8 × 10^2^ ng mL^−1^	–	–	^[^ ^156^ ^]^
IFN‐*γ*	EC	3 pg mL^−1^	10–5 × 10^3^ pg mL^−1^	30 µL	≈60 min	^[^ ^155^ ^]^
TNF‐*α*	EC	–	1–15 pg mL^−1^	–	–	^[^ ^235^ ^]^
IFN‐*γ*	EC	6 pg mL^−1^	10–5 × 10^2^ pg mL^−1^	100 µL	Real time	^[^ ^31^ ^]^
VEGF	EC	0.1 pg mL^−1^	2–5 × 10^2^ pg mL^−1^	–	Real time	^[^ ^126^ ^]^
TNF‐*α*	EC	0.1 pg mL^−1^	0.1–1.5 × 10^2^ pg mL^−1^	–	≈20 min	^[^ ^29^ ^]^
IL‐1*β*, IL‐10	EC	0.3 pg mL^−1^ (IL‐10) 0.7 pg mL^−1^ (IL‐1*β*)	1–15 pg mL^−1^	–	≈45 min	^[^ ^149^ ^]^
TNF‐*α*	EC	0.1 pg mL^−1^ in tears 2 pg mL^−1^ in cerebrospinal fluid and blood serum	1–25 pg mL^−1^	1 µL	–	^[^ ^147^ ^]^
TNF‐*α*	EC	38 pg mL^−1^	0–2.9 × 10^2^ pg mL^−1^	–	≈5 min	^[^ ^236^ ^]^
IL‐6	EC	1.5 pg mL^−1^	4.7–3 × 10^2^ pg mL^−1^	–	Real time	^[^ ^237^ ^]^
TNF‐*α*	EC	–	1–10^2^ pg mL^−1^	–	–	^[^ ^25^ ^]^
IL‐6, TNF‐*α*	EC	20 pg mL^−1^	–	–	Near real time	^[^ ^238^ ^]^
TNF‐*α*	EC	38 pg mL^−1^	76–5 × 10^3^ pg mL^−1^	250 µL	–	^[^ ^239^ ^]^
IL‐1*β*, TNF‐*α*	EC	0.4 pg mL^−1^	1–2 × 10^2^ pg mL^−1^	2.5 µL	≈200 s	^[^ ^153^ ^]^
IL‐3	EC	5 pg mL^−1^	–	100 µL	≈50 min	^[^ ^240^ ^]^
IFN‐*γ*	EC	10 pg mL^−1^	10–10^3^ pg mL^−1^	10 µL	Real time	^[^ ^121^ ^]^
IL‐1, IL‐6, TNF‐*α*	EC	5 pg mL^−1^	5–150 pg mL^−1^	–	–	^[^ ^66^ ^]^
IL‐1*β*	Optoelectronic biosensor	0.3 pg mL^−1^	0.1–10^3^ pg mL^−1^	–	≈10 min	^[^ ^241^ ^]^
TNF‐*α*	Piezoelectric biosensor	1.6 pg mL^−1^	–	50 µL	–	^[^ ^242^ ^]^
IL‐1*β*, IL‐1*α* IL‐6, IL‐10, TNF‐*α*, GM‐CSF	ELISA	0.01–0.03 pg mL^−1^	–	150 µL	≈45 s	^[^ ^243^ ^]^
IFN‐*γ*	ELISA	40 pg mL^−1^	16–2 × 10^3^ pg mL^−1^	–	≈8 min	^[^ ^117^ ^]^
IL‐1*β*, IL‐2, IL‐4, IL‐6, IL‐10, IL‐12*β*, IL‐18, IFN‐*γ*, TNF	PCR	0.03 pg mL^−1^	–	–	–	^[^ ^244^ ^]^
IL‐1*β*, IL‐10 TNF‐*α*,	PCR	10–10^2^ copies	10–10^7^ copies per µL	50 µL	–	^[^ ^245^ ^]^
IL‐2	LoC	50 pg mL^−1^	50–10^3^ pg mL^−1^		≈30 min	^[^ ^112^ ^]^
IL‐6, IL‐8, TNF	LoC	20 pg mL^−1^	–	0.16 µL	15–30 min	^[^ ^113^ ^]^
IL‐10	LoC	1 pg mL^−1^	1–15 pg mL^−1^	3 µL	–	^[^ ^150^ ^]^
TNF‐*α*	Surface‐enhanced Raman spectroscopy (SERS)	4.5 pg mL^−1^	0–10^5^ pg mL^−1^	–	≈2.5 h	^[^ ^141^ ^]^
TNF‐*α*	SERS	1 pg mL^−1^	–	–	–	^[^ ^142^ ^]^
IL‐10	SERS	0.1 pg mL^−1^	0.1–10^2^ pg mL^−1^	–	–	^[^ ^144^ ^]^
IL‐6, VEGF	CLISA	0.05 pg mL^−1^ (IL‐6) 0.03 pg mL^−1^ (VEGF)	0.2–10^2^ pg mL^−1^		–	^[^ ^160^ ^]^
VEGF	Colorimetric sensor	7.4 × 10^3^ pg mL^−1^	–	–	≈60 min	^[^ ^163^ ^]^
VEGF	Colorimetric sensor	4 × 10^3^ pg mL^−1^	4 × 10^3^–1.6 × 10^6^ pg mL^−1^	10 µL	≈60 min	^[^ ^164^ ^]^

#### 4.2.1 Fluorescence Based Cytokines Biosensors

**Figure 5:**
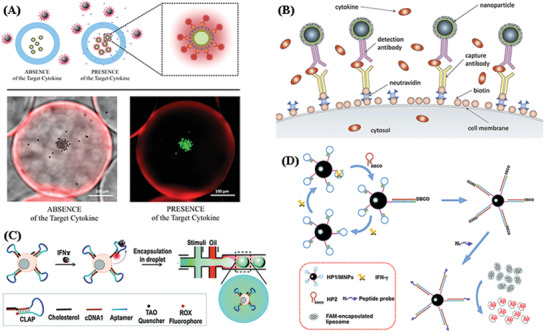
Schematic illustration of A) Sensing with microcapsules. Reproduced with permission.^[^ ^128^ ^]^ Copyright 2019, American Chemical Society. B) An assay where magnetic fluorescent nanoparticles are captured by antibodies on the biotinylated surface of cells. Reproduced with permission.^[^ ^40^ ^]^ Copyright 2019, Elsevier. C) A T cell‐surface aptamer sensor for measuring cytokine secretion at the single‐cell level. Reproduced with permission.^[^ ^130^ ^]^ Copyright 2017, The Royal Society of Chemistry. D) The fluorescent method for IFN‐*γ* detection using three target‐responsive liposomes activated by CHA. Reproduced with permission.^[^ ^131^ ^]^ Copyright 2018, The Royal Society of Chemistry.

A fluorescent biosensor is an assay that operates based on a change in the properties of fluorescence signatures upon interactions with target analytes. It is widely used for both analytical sensing and optical imaging. When the analyte is recognized by its receptor, the fluorescence signal, such as fluorescence intensity, emission wavelength and fluorescence lifetime, can be observed in the form of quenching, enhancement or shift in the fluorescence maxima via different mechanisms (electron transfer (eT), charge transfer (CT), or energy transfer (ET) processes).^[^ ^127^ ^]^ Due to its high sensitivity, fast response time, technical simplicity, varieties in dye selection for multiplexing, and capability to realize on‐site and real‐time detection in an inexpensive manner, fluorescence immunoassays have been one of the most widely employed methods for qualitative and quantitative detection of cytokines. Rahimian et al. reported a microencapsulated fluorescent immunoassay^[^ ^128^ ^]^ (**Figure** **5**A) for detection of IFN‐*γ* and TNF‐*α* in minimally processed blood with a limit of detection of 14.8 and 14.4 × 10^−12^ m for IFN‐*γ* and TNF‐*α*, respectively. Cytokines secreted from leukocytes diffuse into the core of a microcapsule and are captured by antibody‐modified beads residing in the core. The target analyte is detected by staining with secondary fluorescently‐labeled antibody. The fluorescence intensity of encapsulated microbeads is related to its concentration in blood. This encapsulated immunoassay symbolizes a promising strategy for keeping sensing elements operational in a highly complex environment such as blood. To detect cytokine secretion from individual cells by applying a capture technology on the cell membrane, the configuration of on‐cell surface ELISA (OnELISA) is presented in Figure 5B. This has been developed for identifying and selecting high cytokine secreting cells.^[^ ^40^ ^]^ Taking advantage of commercially available magnetic beads labeled with dragon green fluorescence, the OnELISA is a sandwich immunosensor capable of detecting IL‐6 in a single cell level (0.1 pg mL^−1^). These on‐cell surface biosensors provide promising approaches for identifying and selecting high cytokine secretions for applications in regenerative medicine. Avoiding cell internalization of the sensing interface on the cell‐membrane is still a major challenge needing further investigation.

The combination of aptamers in fluorescent biosensors has become a promising assay for the selective and sensitive recognition of cytokines. Hashim et al.^[^ ^129^ ^]^ reported a turn‐on fluorescent aptasensor for detection IFN‐*γ* with a low‐nanomolar *K*_d_ value (33.7 ± 9.5 × 10^−9^ m) at 37 °C. This was prepared by simple labeling of fluorescein at the 3′‐end of a short IFN‐*γ* aptamer. Similar to this turn‐on fluorescence biosensor, Qiu et al.^[^ ^130^ ^]^ developed a cell membrane‐anchored sensor for the detection of IFN‐*γ* at single‐cell level by combining a fluorescent aptamer based sensor and droplet microfluidics (Figure 5C). In their work, the cholesterol‐linked aptamer probe (CLAP) could efficiently anchor onto the cell surface based on hydrophobic interactions between the cholesterol tail and the cellular phospholipid layer. Thus, the fluorescence of the aptamer probe could be turned on in the presence of IFN‐*γ*. Finally, aptamer‐decorated T cells can be individually encapsulated into droplets by microfluidic chip systems, enabling the detection of cytokine secretion at the single‐cell level with a sensitivity of 15 ng mL^−1^ (1.5 × 10^4^ pg mL^−1^).

The analytical methods based on single target‐aptamer interactions always lack sensitivity for clinical use. Thus, introducing signal amplification strategies in aptamer‐based biosensors is of great importance. Cui et al.^[^ ^131^ ^]^ proposed a novel fluorescent assay to detect the tuberculosis‐related cytokine IFN‐*γ* by combining DNA self‐assembly based signal amplification with liposome‐based signal amplification, offering a high sensitivity of 0.047 × 10^−12^ m (0.068 pg mL^−1^). The principle of this fluorescence‐based biosensor is illustrated in Figure 5D. Firstly, the sensing hairpin probe (HP) HP1 containing the sequence of the IFN‐*γ* aptamer is immobilized onto the surface of magnetic nanoparticles (MNPs). Another DNA hairpin probe, the signaling hairpin probe HP2, is available for hybridization with HP1 and is tethered by a dibenzocyclooctyne (DBCO) group. Without target IFN‐*γ*, the HP1 and HP2 probes maintain their stem‐loop structures. Once target IFN‐*γ* is present, the combination of IFN‐*γ* and the aptamer region (blue) causes conformational change to HP1 and exposes the single‐stranded sequence (green) that is partially complementary to HP2, promoting strand displacement to form an HP1/HP2 duplex and of release IFN‐*γ* from the aptamer region. Then, the released IFN‐*γ* is free to interact with another intact HP1, initiating a new cycle of catalytic hairpin assembly (CHA). After numerous cycles, a large amount of HP1/HP2 duplexes are produced on the MNPs surface. Finally, an azido‐labeled peptide probe is conjugated to the MNP surface through click chemistry which can destroy the liposome membrane and promote the leakage of fluorescence molecules, realizing the highly sensitive detection of IFN‐*γ*.

In addition to extracellular cytokine detection, fluorescence biosensors have been applied to intracellular cytokine detection. For instance, a simple and sensitive “switch‐on” nanosensor based on graphene quantum dots (GQDs) for the intracellular detection of IFN‐*γ* has been developed that offers a sensitivity of 2 pg mL^−1^.^[^ ^33^ ^]^ The self‐quenching of aggregated GQDs turns off the fluorescence and the disaggregation of GQDs induced by the presence of the target analyte IFN‐*γ* results in fluorescence recovery that is proportional to the concentration of IFN‐*γ*. These fluorescent nanosensors were successfully used for the detection of intracellular IFN‐*γ* in live PBMCs and BV2 cells as basic models, and can be used as universal switch‐on sensing probes targeting a spectrum of intracellular cytokines. Taking advantage of the property of aggregation induced emission agents, a fluorescent aptasensor for the measurement of intracellular IFN‐*γ* secreted by live cells was reported, with a low detection limit of 2 pg mL^−1^ under in vitro conditions.^[^ ^39^ ^]^ This aptasensor consists of a fluorogen (TPEN3) that shows strong red emission only in the presence of IFN‐*γ* and an oligonucleotide which has a high affinity to IFN‐*γ*. The probe is able to localize the intracellular IFN‐*γ* at a low concentration, and it was successfully used for real‐time imaging showing excellent cellular permeability and biocompatibility as well as low cytotoxicity. Consequently, fluorescence based biosensors offer the advantages of sensitivity. They are also rapid response, non‐destructive and real‐time for cytokine detection. However, the major disadvantage of using fluorescence‐based optical biosensors is background interference and the requirement for sample labeling with fluorescent reagents which adds time and cost to the procedure.

#### 4.2.2 Surface Plasmon Resonance Based Cytokines Biosensors

**Figure 6:**
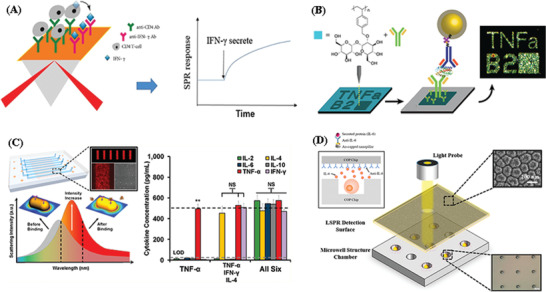
Schematic illustration of A) CD4+ T‐cell capture and real‐time monitoring of IFN‐g release. Reproduced with permission.^[^ ^136^ ^]^ Copyright 2018, Taylor & Francis Group. B) Direct protein patterns for multiplexed cytokine detection. Reproduced with permission.^[^ ^137^ ^]^ Copyright 2016, American Chemical Society. C) LSPR microarray chip. Reproduced with the permission.^[^ ^24^ ^]^ Copyright 2015, American Chemical Society. D) Integrated localized surface plasmon resonance (LSPR) cytokine detection. Reproduced with permission.^[^ ^138^ ^]^ Copyright 2020, MDPI.

Sensing using SPR is widely used for implementing biosensing in clinical analysis as it provides a label‐free and real‐time format to measure biomolecular interactions.^[^ ^132^ ^]^ The basic principle of SPR biosensors has been reviewed by Guo^[^ ^133^ ^]^ In SPR systems, the analyte is captured by biomolecular recognition elements on the metal surface of a SPR biosensor, changing the refractive index at the metal surface. The changes of refractive index can then be accurately measured by different optical means such as intensity modulation, angular modulation, wavelength modulation, phase modulation, and even polarization modulation. As an advantage, the concentration of analytes can be monitored continuously by measuring the spectral shift of the resonance dip without additional labels. SPR‐based biosensors^[^ ^134^, ^135^ ^]^ have been successfully applied to measure cytokines for the diagnosis of diseases, due to their sensitivity and ability to perform label‐free measurement in real time. For instance, Wu et al. reported a label‐free SPR biosensor for real‐time monitoring of captured human CD4+T‐cells, and their dynamic IFN‐*γ* production (**Figure** **6**A),^[^ ^136^ ^]^ enabling the diagnosis of tuberculosis (TB) in clinical samples with high sensitivity (85.5%) and specificity (97.7%). The CD4+‐ T cells were captured by anti‐CD4 Abs, and the culture media containing the TB‐specific proteins was injected to stimulate the captured CD4+ T cells to release IFN‐*γ*. SPR signals were monitored in real‐time when adding TB‐specific proteins, allowing for quantification of IFN‐*γ* protein secreted by CD4+ cells. Lau et al.^[^ ^137^ ^]^ fabricated a localized surface plasmon resonance (LSPR) immunoassay (Figure 6B) for the detection of secreted cytokines (IL‐6 and TNF‐*α*) from stimulated macrophages utilizing electron beam lithography and a trehalose glycopolymer for the direct writing of antibodies on silicon substrates. This sandwich immunoassay was visualized via dark‐field microscopy, exploiting the surface plasmon resonance of silver‐enhanced gold nanoparticle secondary antibodies. Multiplexing measurement of IL‐6 and TNF‐*α* on a single chip was also successfully demonstrated with high specificity and sensitivity (5 pg mL^−1^ for TNF‐*α* and 50 pg mL^−1^ for IL‐6). This direct fabrication of capture antibody patterns for cytokine detection could be useful for biosensing applications.

Chen et al. developed a label‐free, multiarrayed localized SPR (LSPR)‐based optical biosensor chip (Figure 6C) for massively parallel high‐throughput detection of multiple cytokines (IL‐2, IL‐4, IL‐6, IL‐10, IFN‐*γ*, TNF‐*α*).^[^ ^24^ ^]^ The device was fabricated using easy‐to‐implement, one‐step microfluidic patterning and antibody conjugation of gold nanorods (AuNRs). The nanorod microarray fabrication was performed using a one‐step microfluidic patterning technique assisted by electrostatic attractive interactions between the nanorods and the substrate surface within microfluidic channels. Subsequently, these nanorod microarrays were integrated in a microfluidic chip with eight parallel microfluidic detection channels consisting of inlet and outlet ports for reagent loading and washing. Specific antibodies were conjugated to the patterned AuNR microarrays using thiolated crosslinker and EDC/NHC chemistry. The current chip design integrates 480 AuNR microarray sensor spots. The prepared LSPR microarray chip was then imaged under dark‐field microscopy and scanning electron microscopy (SEM). This LSPR biosensing technique allowed for high‐sensitivity quantitative cytokine measurements at concentrations down to 5–20 pg mL^−1^ from a 1 µL serum sample. Zhu et al.^[^ ^138^ ^]^ reported the simple synergistic integration of cell trapping of the microwell chip and gold‐capped nanopillar‐structured cyclo‐olefin‐polymer (COP) films using LSPR technology for IL‐6 detection (Figure 6D) with sensitivity of 190.2 nm RIU^−1^ and detection limit of 10 ng mL^−1^. In this research, fresh cultured IL‐6 over‐expressed Jurkat cells were utilized to evaluate the sensitivity and capability of this LSPR based biosensor. The cultured cells were directly trapped by thick COP cell trapping chips and started to release IL‐6 which would immediately bind with the antibody on the surface of the nanopillar‐structured LSPR detection film without stimulation. The fabricated device shows the potential for real‐time monitoring of cytokines which would allow one to identify the viability and biological variation of the tested single cell. Although SPR has wide applications for sensing proteins, a common challenge with SPR‐based sensors is the issue of signals produced via non‐specific binding events on the sensor. This is an issue that requires more investigation by applying strategies for avoiding anti‐fouling.^[^ ^139^ ^]^

#### 4.2.3 Surface Enhanced Raman Spectroscopy Based Cytokine Sensors

**Figure 7:**
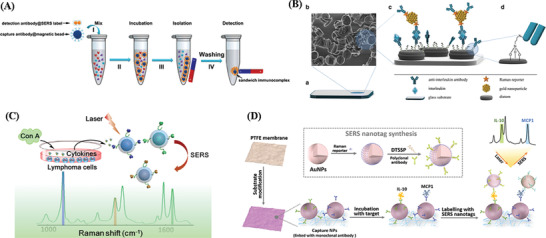
Schematic illustration of A) Multiplexed SERS nanotags for the detection of cytokines secreted by lymphoma. Reproduced with permission.^[^ ^142^ ^]^ Copyright 2018, Springer. B) Multiplexed SERS nanotags for the detection of cytokines secreted by a lymphoma. Reproduced with permission.^[^ ^143^ ^]^ Copyright 2017, Springer. C) Multiplexed SERS nanotags for the detection of cytokines secreted by a lymphoma. Reproduced with permission.^[^ ^141^ ^]^ Copyright 2019, American Chemical Society. D) The workflow of the assay on the paper‐based substrate for MCP‐1 and IL‐10 duplex detection. Reproduced with the permission.^[^ ^144^ ^]^ Copyright 2019, The Royal Society of Chemistry.

SERS has become a powerful vibrational spectroscopy technique that allows for high‐sensitivity detection of low concentration analytes through the amplification of electromagnetic fields generated by the excitation of localized surface plasmons^[^ ^140^ ^]^ SERS based biosensors, a popular and promising assay, has been widely used for the fast and quantitative measurement of cytokines owing to its outstanding features such as high sensitivity, high specificity and multiplexed non‐destructive detection capability.^[^ ^141^ ^]^ Lai et al. used a magnetic bead pull‐down assay combined with SERS^[^ ^142^ ^]^ (**Figure** **7**A) for the rapid and sensitive detection of TNF‐*α*. The stable, monodisperse, and highly sensitive SERS labels were fabricated by purified silica‐encapsulated small AuNP clusters. Silica encapsulation improves the stability of SERS labels for reproducible signals and offers a robust surface for subsequent bioconjugation providing high specificity, selectivity and sensitivity of 1 pg mL^−1^ for TNF‐*α* measurement. The characteristic Raman peaks and barcode signals from up to three different Raman reporters in colloidal mixtures could be identified, indicating the great potential of these SERS labels as sensitive reporters in multiplexed bioanalytical applications. Kamińska et al. developed a SERS immunoassay^[^ ^143^ ^]^ based on diatom biosilica as the immune substrate and gold nanoparticles (AuNPs) functionalized with DTNB (i.e., 5,5′‐dithiobis(2‐nitrobenzoic acid)) as the Raman reporter for the detection of IL‐8 in blood plasma. These DTNB‐labeled immune‐AuNPs can form a sandwich structure with IL‐8 antigens and the antibodies immobilized on the biosilica material; this is illustrated in Figure 7B. The established SERS immunoassay with lower detection limit of 6.2 pg mL^−1^ offers a valuable platform for the ultrasensitive and highly specific detection of cytokines in a clinical setting.

The narrow SERS vibrational bands promise multiplex capability that can produce a unique fingerprint of the cytokine network in a single test by using different SERS nanotags. Thus, efforts have been devoted to the development of SERS‐based biosensors for sensitive and multiplexed cytokine detection in different diseases.^[^ ^144^ ^]^ For sensitive and simultaneous detection of multiple cytokines, Li et al. developed SERS nanotags (Figure 7C) composed of a gold core, Raman reporter cells, and a silver shell, which has been used for sensitive and multiplexed identification of cytokines (IFN‐*γ*, TNF‐*α*, and IL‐10) secreted from lymphoma cells.^[^ ^141^ ^]^ This SERS immunoassay showed high sensitivity (4.5 pg mL^−1^) with good specificity. More importantly, this sandwiched immunoassay strategy was much faster in response than many traditional approaches for its multiplex capability, while its detection limit and accuracy were comparable to those of the standard ELISA assay. The identification of three cytokines, IFN‐*γ*, TNF‐*α*, and IL‐10, secreted from the lymphoma cell lines upon Con A stimulation further demonstrated the potential of the proposed assay for clinical diagnosis. In another work and for atherosclerosis (AS) associated disease diagnosis, a paper‐based SERS assay (Figure 7D) was reported for sensitive duplex cytokine (IL‐10 and MCP‐1) detection.^[^ ^144^ ^]^ This SERS biosensor combines a nanoporous networking membrane by utilizing a polymer membrane fabricated from a polypropylene (PP) substrate with a polytetrafluoroethylene (PTFE) coating as the substrate and SERS nanotags as the probe for signal detection, together with a sandwich design. It demonstrated sensitive and specific identification and quantification of cytokines targets in human serum with excellent sensing characteristics. In this work, the increased surface area offered high loading of capture antibodies which enhanced the sensitivity. Due to its unique feature of two‐layer AuNPs in the sandwich design, a small gap was generated between the AuNPs; this produced a “hot‐spot” effect that could enhance the SERS signal, allowing high sensitivity detection with a low detection limit (0.1 pg mL^−1^). Therefore, paper‐based SERS assay platforms can be potentially used for cytokines detection, even as commercial units, and holds great potential for applications in complicated environments for multiplexed target analysis.

#### 4.2.4 Electrochemical Based Cytokine Biosensors

**Figure 8:**
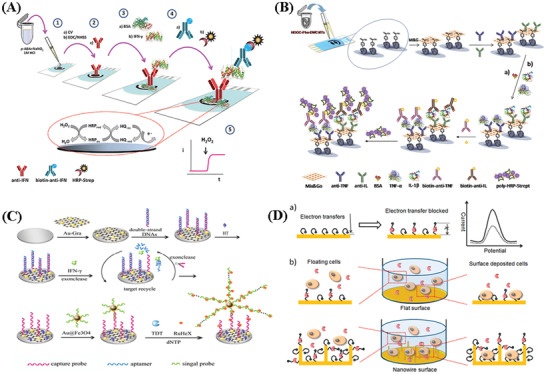
Schematic illustration of A) Steps involved in the preparation of the electrochemical immunosensor for the determination of IFN‐*γ*. Reproduced with the permission.^[^ ^152^ ^]^ Copyright 2020, Elsevier. B) The different steps involved in the preparation of the dual electrochemical immunosensor for multiplexed determination of IL‐1*β* and TNF‐*α*. Reproduced with permission.^[^ ^153^ ^]^ Copyright 2016, Elsevier. C) The stepwise aptasensor fabrication based on exonuclease‐catalyzed target recycling and surface‐initiated enzymatic polymerization for amplification. Reproduced with the permission.^[^ ^155^ ^]^ Copyright 2015, The Royal Society of Chemistry. D) An aptamer‐based electrochemical sensor for IFN‐*γ*. Reproduced with the permission.^[^ ^156^ ^]^ Copyright 2017, American Chemical Society.

Electrochemical transduction is very popular as a biosensor technology. Compared with other methods, electrochemical techniques have their own advantages, such as low cost, high sensitivity particularly in amperometric based measurements, and the possibility of facile device miniaturization.^[^ ^145^, ^146^ ^]^ The output of electrical signals can be impedance, current, and voltage. Many electrochemical biosensors have been developed for the detection of cytokines.^[^ ^147^, ^148^, ^149^, ^150^ ^]^ Filik et al.^[^ ^151^ ^]^ summarized the recent developments of numerous electrochemical assays for the measurement of TNF‐*α*, illustrating various novel sensing strategies for immunoelectrochemical sensor improvement to selectively detect cytokines. Sanchez‐Tirado et al.^[^ ^152^ ^]^ reported a simple and sensitive amperometric immunosensing assay taking advantage of the great performance of grafted electrochemical scaffolds for covalent immobilization of biomolecules for the detection of IFN‐*γ* in saliva. **Figure** **8**A shows a schema of the steps for the fabrication of this electrochemical immunosensor as well as the reactions involved in the amperometric detection. The screen‐printed carbon electrode (SPCE) was functionalized by grafting of the diazonium salt of *p*‐aminobenzoic (*p*‐ABA) by cyclic voltammetry (step 1) for the covalent immobilization of the capture antibody (step 2), and the remaining free active sites were blocked with BSA (step 3). After capture of IFN‐*γ*, a sandwich‐type immunoassay was implemented using biotin‐anti‐IFN and peroxidase‐labeled streptavidin (HRP‐Strept) (step 4). Amperometric measurements were carried out by adding hydrogen peroxide solution to the electrode surface in the presence of hydroquinone (HQ) as the redox mediator (step 5), obtaining a low limit of detection of 1.6 pg mL^−1^ for IFN‐*γ* quantification. The analytical performance displayed by this electrochemical immunosensor including disposability and the possibility of using pocket‐sized electrochemical instrumentation makes it attractive for the development of POC systems for on‐site measurement of salivary IFN‐*γ*. In another development electrochemical nanosandwich devices based on a graphene oxide (GO) thin film modified sensing interface was fabricated for the detection of IL‐6.^[^ ^30^ ^]^

To realize the measurement of multiple cytokines via electrochemical assays, Shen et al. fabricated label‐free electrochemical biosensors for in vivo cytokine detection of multiple cytokines in a Parkinson's disease mice model.^[^ ^66^ ^]^ Wei et al. also developed an electrochemical immunosensor^[^ ^28^ ^]^ for the simultaneous detection of three cytokines IL‐6, IL‐1*β*, and TNF‐*α*. The glassy carbon (GC) surface was functionalized by attaching mixed layers of 4‐carboxylic phenyl and 4‐aminophenyl phosphorylcholine (PPC) as the sensing interface for immobilization of the capture monoclonal antibodies for IL‐6, IL‐1*β*, and TNF‐*α*. After capturing IL‐6, IL‐1*β* and TNF‐*α*, GO loaded with redox probes (Nile blue (NB), methylene blue (MB), or Ferrocene (Fc)) and anti‐cytokine antibodies for the specific cytokines were introduced. The quantitative detection of the cytokines was achieved by monitoring the change in electrochemical signals from signal reporters. This system was successfully used for detection simultaneously with desirable performance in sensitivity, selectivity, stability, and recovery. Sanchez‐Tirado et al. developed an electrochemical immunosensor using dual SPCE functionalized with double‐walled carbon nanotubes for simultaneous detection of IL‐1*β* and TNF‐*α* in serum and saliva.^[^ ^153^ ^]^ The scheme for the preparation of the dual electrochemical immunosensor is shown in Figure 8B. After dropping Mix&GO onto each surface of the dual SPCE, antibodies to IL‐1*β* and TNF‐*α* were immobilized, following with a blocking step of the remaining active free sites on the electrode surfaces via BSA. Then, sandwich type assays were implemented by combining the target cytokines and biotinylated detector antibodies. A further conjugation with poly‐HRP‐Strept allowed the amperometric determination of IL‐1*β* and TNF‐*α* using H_2_O_2_ as HRP substrate and HQ as the redox mediator.

Electrochemical immunoassays have seen great improvements and they are expected to make significant contributions in future studies of disease pathologies. For immunosensors, structure‐switching aptamer based electrochemical biosensors can also be implemented to realize real‐time cytokines detection.^[^ ^31^, ^36^, ^38^ ^]^ Specifically, in order to enhance the sensitivity and robustness of aptamer based cytokine biosensing, the surface nanofabrication and ratiometric measurements are important.^[^ ^126^, ^154^ ^]^ Liu et al. developed an aptamer‐based electrochemical immunosensor (Figure 8C) depending on exonuclease‐mediated surface‐initiated enzymatic polymerization (SIEP) combined with [Ru(NH_3_)_6_]^3+^ for IFN‐*γ* detection.^[^ ^155^ ^]^ First, the electrode surface was functionalized with gold nanoparticles‐graphene nanocomposite (Au‐Gra). Then, the hybridized double‐stranded DNAs (dsDNA) were immobilized on the modified electrode surface following with a block of non‐specific sites by hexanethiol solution (HT). After adding IFN‐*γ*, the aptamer was liberated from dsDNA, and selectively digested by the RecJf exonuclease, making IFN‐*γ* released for target recycling. After that, a great number of single‐stranded capture probes were formed during the cyclic process. Subsequently, numerous signal probe‐labeled Au@Fe_3_O_4_ (SP‐Au@Fe_3_O_4_) were captured by single‐stranded capture probes on the electrode surface. Finally, the labeled signal probe sequences were catalyzed by the terminal deoxynucleotidyl transferase (TdT)‐mediated cascade extension to form a long ssDNA structure for electrostatic adsorption of [Ru(NH_3_)_6_]^3+^, generating the electrochemical signal. This proposed aptasensor displayed a low detection limit of 0.003 ng mL^−1^, providing a simple, sensitive, and powerful tool for the reliable detection of IFN‐*γ* and other cytokines. Furthermore, the proposed sensor has potential for clinical diagnostics, infectious disease monitoring, and point‐of‐care testing. Ni et al. developed a robust aptasensor^[^ ^154^ ^]^ by modifying methylene blue loaded graphene oxide (GO/MB) and ferrocene‐labeled aptamer onto GC electrodes to realize the dual electrochemical signal mode ratiometric quantifications of vascular endothelial growth factor (VEGF) in serum with wider linear range (10–5 × 10^2^ pg mL^−1^) and better sensitivity (10 pg mL^−1^). The advantages of large nanostructured surfaces in aptamer‐based electrochemical biosensors was shown by Liu et al. to result in more sensitive analysis of cell‐secreted cytokines, by improved transport and enhanced surface area per footprint. Liu et al employed silicon nanowires (Si NWs) covered with gold as working electrodes for aptamer‐based detection of IFN‐*γ*.^[^ ^156^ ^]^ Aptamer molecules were designed to form a hairpin structure and the redox reporter molecule methylene blue was in close proximity to the electrode surface (Figure 8D, a). Binding of IFN‐*γ* caused the redox label to move further away from the electrode by changing the conformation of the hairpin and inhibited electron transfer from redox reporters, decreasing the electrochemical redox signal. The differences in the faradaic current before and after IFN‐*γ* binding were quantified using square wave voltammetry (SWV). Figure 8D, b) shows the different behavior of cell deposition and cytokine IFN‐*γ* secretion for floating cells and surface deposited cells on planar Au electrode and the AuNWs electrode. A series of experiments demonstrated that NW aptasensors responded faster and were more sensitive to IFN‐*γ* compared to standard flat electrodes, allowing measurement of IFN‐*γ* with a low detection limit of 0.14 ng mL^−1^. This NW aptasensor possesses a larger surface area and higher aptamer packing density and is minimally affected by the direct deposition of leukocytes. It shows great potential for cytokine detection and addresses the important need for sensitive diagnosis of diseases.

#### 4.2.5 Other Types of Cytokine Biosensors

**Figure 9:**
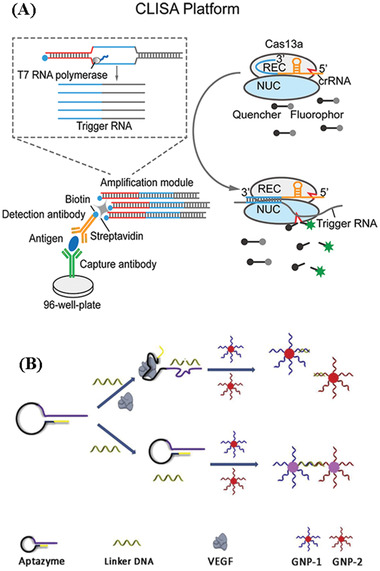
Schematic illustration of A) The CRISPR/Cas13a signal amplification system. Reproduced with the permission.^[^ ^160^ ^]^ Copyright 2019, American Chemical Society. B) The proposed method for protein detection, utilizing VEGF as an example. Reproduced with the permission.^[^ ^164^ ^]^ Copyright 2016, Elsevier.

The CRISPR/Cas (clustered regularly interspaced short palindromic repeats/CRISPR associated proteins) biosensing is a highly sensitive and selective tool for detection of different targets including cytokines.^[^ ^157^, ^158^ ^]^ In addition to Cas9 and Cas12 factors, the recent discovery of the collateral RNA cleavage activity of the Cas13a effector has attracted greater attention to develop novel biosensing technologies for nucleic acid detection.^[^ ^159^ ^]^ CRISPR/Cas13a also enables the development of direct RNA assays with high sensitivity for cytokine detection. Chen et al.^[^ ^160^ ^]^ reported a CRISPR/Cas13a signal amplification linked immunosorbent assay (CLISA) for the detection of IL‐6 and VEGF. This assay (**Figure** **9**A) double‐amplifies the output signal by T7 RNA polymerase transcription and CRISPR/Cas13a collateral cleavage activity. T7 polymerase can recognize the promoter sequence to perform the transcription, and many copies of single‐stranded RNA molecules are produced. The CRISPR/Cas13a system enables to recognize the transcribed RNA molecules accurately, leading to the activation of trans‐cleavage activity of CRISPR/Cas13. Short single‐stranded RNA reporter labeled with fluorophore and quencher groups at both ends of the sequence in the system can be cleaved by the transcleavage activity, generating fluorescent signal. This CRISPR/Cas13a biosensing possessed high sensitivity and achieved lower detection limits of femtomolar level for cytokine measurement, which allows for rapid screening of large numbers of samples simultaneously, providing potential ultrasensitive detection methods for biosensing, medical research, and molecular diagnostics.

Colorimetric sensors^[^ ^161^, ^162^ ^]^ based on color changes have attracted attention for the instantaneous detection of various analytes owing to its intrinsic advantages, such as the simple processing and visual indication. The optical property of noble metal nanoparticles provides a visual color change when they interact with the analyte due to the dispersion and aggregation of nanoparticles.^[^ ^161^ ^]^ Consequently, noble metal nanoparticles have potential for biomolecular detection in colorimetric sensing. Gold nanoparticles (AuNPs) are one of the most popular noble metal entities in colorimetric assay for cytokine detection. For example, Chang et al.^[^ ^163^ ^]^ reported an aptamer‐based colorimetric biosensor using AuNPs combined with a branched DNA amplification strategy for the quantification of VEGF, an important cytokine for angiogenesis and vascular permeabilization. Using this colorimetric biosensor, as few as 3.7 fmole of VEGF could be detected within an hour. Wu et al.^[^ ^164^ ^]^ proposed a colorimetric sensor for VEGF measurement based on target‐triggered activation of aptazyme with AuNPs. As shown in Figure 9B, an aptazyme consisting of a VEGF aptamer (black), DNAzyme (purple) and a short stem sequence (yellow) is designed for this assay. Additionally, a linker is designed to contain the sequence to crosslink AuNPs and to have the substrate sequence of the DNAzyme. Therefore, in the presence of VEGF, the recognition between VEGF and its aptamer causes the conformational change of the aptazyme which activates the DNAzyme. Thus, the color of AuNPs solution remains red since the AuNPs cannot be crosslinked. Nevertheless, in the absence of the target protein, the color change will happen. Based on the change of the color, a simple, rapid, and cost‐effective method was developed for VEGF quantification with as low as 0.1 × 10^−9^ m detection limit.

Microring resonator (MR) sensing, based on the accurate measurement of changes in the index of refraction of the receptor layer toward surface binding between a target and antibody‐modified microrings, has found numerous applications for the label‐free and real‐time detection of biomolecules.^[^ ^165^, ^166^, ^167^, ^168^ ^]^ The advantages of this MR based sensor include high mechanical stability, detection sensitivity, scalability to sensor networks, and cost reduction due to wafer scale processing.^[^ ^169^ ^]^ Thus, this method represents a promising platform for real‐time detection of cytokines. Silicon photonic microring resonators, a class of high‐Q optical microcavities, have recently shown promise for label‐free bioanalysis of cytokines due to real‐time reaction monitoring capabilities, high scalability on a small footprint, low per‐device cost, and ease of fabrication.^[^ ^170^ ^]^ For example, Kindt et al. reported a multiplexable silicon photonic MR platform integrated with an enzymatic signal enhancement scheme for simultaneous quantification of IL‐2, IL‐6, and IL‐8 in undiluted cerebrospinal fluid, providing a limit of detection at or below 1 pg mL^−1^.^[^ ^171^ ^]^ This research has shown that MR based sensing platforms have significant potential for multiplexed cytokine measurement at ultralow concentrations. In addition, silicon photonic MR platforms combined with sandwich immunoassays can achieve real‐time monitoring of multiple cytokines. Luchansky and Bailey realized this simultaneous detection of four cytokines (IL‐2, IL‐4, IL‐5, and TNF‐*α*) secreted from primary human T cell populations using only a 5 min assay performed on an intrinsically scalable silicon photonic MR analysis platform.^[^ ^170^ ^]^ Altogether, MR based biosensors are promising platforms for a number of multiplexed and real‐time in vitro diagnostic applications and should be investigated further.

The interferometric reflectance imaging sensor (IRIS) is another potential technique for label‐free and real‐time detection of cytokines. In this biosensing process, transduction is based on spectral reflectivity. As the overall thickness of the upper layer is increased due to biomass accumulation on the surface of the layered substrate, the optical path difference (OPD) between the top surface of the substrate and the silicon substrate covered with a layer of silicon diooxide (Si‐SiO_2_) interface also increases; this in turn results in a quantifiable shift in spectral reflectivity.^[^ ^172^ ^]^ Thus, this functional platform allows for accurate, label‐free and dynamic monitoring of surface bound biomolecules. The utility of the IRIS technique has been demonstrated in the real‐time measurement of IL‐6 in cell culture medium.^[^ ^173^ ^]^ The label‐free biosensor resulted in more than sevenfold signal improvement for detecting IL‐6 with the expected limit of detection approaching 2 ng mL^−1^. Therefore, IRIS can be used as a platform for in vitro analysis of an immunological response or to monitor disease progression.

### 4.3 Commercial Cytokine Detection Assays

As we have discussed in previous sections, quantification of multiple cytokines in clinically relevant samples is essential in biology and medicine. In addition, ELISAs are available to detect the single cytokine and a number of commercial multiplex technologies (Luminex‐based or flow cytometry (FCM)‐based) are available. Most of these assays, based on fluorescent bead‐based technology allows the profiling of multiple cytokines in a small volume.^[^ ^174^ ^]^ These commercial multiplex assays possess more advantages^[^ ^175^ ^]^ than singleplex assays (e.g., ELISA), including 1) small sample volume requirement, 2) reduction in assay time, and 3) a larger range of quantification for each analyte. This section introduces three main commercial cytokine detection assays, Luminex assays, flow cytometry and mesoscale discovery (MSD) assays by discussing performance (**Table** **4**), principles, and applications.

**Table 4:** Performance of the commercial cytokine kits

Kit name	Platform	Sample media	Cytokine sources	Number of cytokines	Signal readout	Linear range [pg mL^−1^]
Bio‐Plex Pro assay	Luminex	Tissue and cell culture supernatants, plasma, and serum	Human	27	Median fluorescence intensity (MFI)	10–10^4^
MILLIPLEX MAP plex kit	Luminex	Human serum, plasma and cell culture supernatants	Human	13	MFI	3.2–10^4^
Invitrogen 25‐plex kit	Luminex	Human serum, plasma and cell culture supernatants	Human	25	MFI	0.5–3.6 × 10^4^
Invitrogen magnetic 30‐plex kit	Luminex	Human serum, plasma and cell culture supernatants	Human	30	MFI	0.5–3.6 × 10^4^
Human enhanced sensitivity 3‐plex kit	Flow cytometry	Tissue culture supernatants, plasma, and serum samples	Human	3	MFI	0.3–2 × 10^2^
Becton Dickinson human T_h_1/T_h_2 cytokine kit	Flow cytometry	Tissue culture supernatants, EDTA plasma, and serum samples	Human	14	MFI	Nondetermined −5 × 10^3^
MACSPlex cytokine kit	Flow cytometry	Serum, plasma, and cell culture supernatants	Human	12	MFI	Nondetermined −10^4^
Plex Th cytokine 13‐plex panel	Flow cytometry	Serum and cell culture supernatant samples	Human	13	MFI	Nondetermined −10^4^
U‐PLEX biomarker group 1 (NHP) assays	MSD	Serum, plasma, cell culture supernatant	Nonhuman primates	30	Light signal	Nondetermined −1.8 × 10^5^
Human T_h_1/T_h_2 10‐plex Ultra‐Sensitive kit	MSD	Serum, plasma	Human	10	Light signal	Nondetermined −10^4^
Human pro‐inflammatory‐9 ultrasensitive kit	MSD	Serum, plasma	Human	9	Light signal	Nondetermined −10^5^

#### 4.3.1 Luminex Assays

Luminex assays have become increasingly important for the detection and quantification of multiple cytokines due to its capacity to measure many cytokines simultaneously in a single assay with a small sample volume requirement.

**Figure 10:**
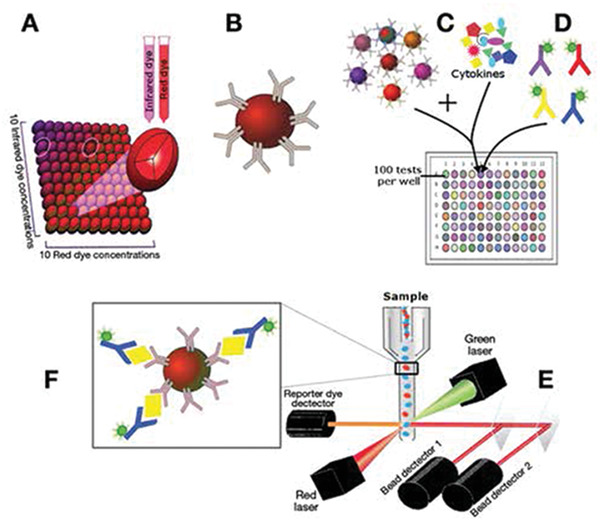
Luminex technique and general principles. Reproduced with the permission.^[^ ^176^ ^]^ Copyright 2015, The Society for Investigative Dermatology.

The Luminex system^[^ ^176^, ^177^ ^]^ enables fast and accurate measurements of cytokines based on utilizing hundreds of microsphere or bead sets marked with differing ratios of two different fluorophores (**Figure** **10**A) conjugated with monoclonal antibodies specific for different cytokines (Figure 10B). When cytokines (Figure 10C) have bound, secondary detection antibodies (Figure 10D) for the specific cytokines are added. The detection antibodies are conjugated with signal dyes, providing the microsphere with an additional distinct fluorescent emission signature upon binding the cytokine (Figure 10F). Then the beads are read on a Luminex machine (Bioplex‐100, Bio‐Rad) which has two lasers (Figure 10E) for the identification of the bead and for quantifying the detection agent on the beads respectively, thus realizing quantification of multiple cytokines in a single sample.

Applications of Luminex cytokine assays have rapidly expanded for monitoring of immunity in clinical trials, especially for early diagnosis of many diseases. For example, Koshiol et al.^[^ ^178^ ^]^ evaluated the performance of the Luminex assay for quantifying various cytokines and other biomarkers in cervical secretions; the results provided initial evidence for possible associations between those markers and progression of HPV‐associated cervical pre‐cancers. Troy et al.^[^ ^175^ ^]^ measured cytokines in bile obtained from gallstone patients utilizing Luminex and studied the role of immune processes in gallbladder‐related diseases. Thus, this technology enables measurements of numerous cytokines within and between experiments providing a more inclusive and comprehensive depiction of disease than the detection of individual cytokines. However, several factors^[^ ^176^ ^]^ may limit the utility and availability of Luminex, such as the dedicated requirement for analysis instruments and the upfront costs. Luminex assays are also subject to variability because of assay manufacturer, product lot number, and assay execution.

#### 4.3.2 Flow Cytometry

The measurement of intracellular cytokines has a significant impact on the way that immune function is assessed. FCM^[^ ^179^, ^180^ ^]^ based on staining of cytokines and cell surface markers with specific fluorescence‐labeled antibodies is a highly effective assay for the detection of cytokines. This technology permits simultaneous detection of multiple cytokines at the single cell level with high throughput, thus offering a tremendous advantage over other single‐cell methods.

The main steps in the FCM assay^[^ ^179^ ^]^ are cell collection, fixation, permeabilization, blocking, intracellular staining, and analysis by FCM. Cells are treated with protein secretion inhibitors (monensin or brefeldin A) to block intracellular cytokine transport and then digested with tryptase. Subsequently, paraformaldehyde fixation is applied for the stabilization of cell membranes and for preservation of intracellular antigenicity of the cytokines, as well as for enabling the cells to withstand permeabilization by a detergent. Then, the cell membranes are permeated using the detergent to permit the cytokine‐specific monoclonal antibodies to penetrate the cell membrane, cytosol, and membranes of the endoplasmic reticulum and Golgi apparatus. Next, a blocking step is necessary before intracellular cytokine staining to block the nonspecific binding of the antibodies which reduces nonspecific staining and improves the specificity of detection. Staining is based on the utilization of specific fluorescence‐labeled antibodies for intracellular cytokines. Finally, samples are measured by FCM, revealing the size and fluorescence intensity of the cells, and the populations that express the target cytokine.

Traditionally monensin or brefeldin A (BFA) are used as protein transport inhibitors, to inhibit cytokine transport. This results in the accumulation of the cytokine of interest intracellularly.^[^ ^181^ ^]^ The distinct mechanism^[^ ^182^ ^]^ of both inhibitors to prevent intracellularly produced proteins is well known. In FCM, it is necessary to discuss the influence of the chosen protein transport inhibitor on cytokine detection. In general, monensin^[^ ^183^ ^]^ is more toxic than BFA when incubation periods exceed 18 h. To compare the capacity of cytokine secretion inhibitors, Schuerwegh et al.^[^ ^182^ ^]^ evaluated the efficiency of both monensin and brefeldin A in inhibiting the cytokine secretion of peripheral blood monocytes in rheumatoid arthritis patients and healthy persons. The study found that, for flow cytometric detection of intracellular monocytic cytokines (IL‐1*β*, IL‐6, and TNF‐*α*), brefeldin A was a more potent, effective, and less toxic inhibitor of cytokine secretion than monensin. More research about monensin or brefeldin A on the influence of cytokine measurement was reported by Muris et al.^[^ ^181^ ^]^ and Miguel and co‐workers.^[^ ^183^ ^]^

The flow cytometric assay based on intracellular cytokine staining is the primary immunological technique for evaluating vaccine efficacy in clinical trials. This technique allows for the measurement of antigen‐specific T cell function, specific cytokines and cell surface markers expression.^[^ ^180^ ^]^ This technology permits identification of antigen‐specific T cells induced in response to the proteins expressed in a candidate vaccine. De Rosa^[^ ^184^ ^]^ reported the use of flow cytometry for the measurement of several cytokines in HIV vaccine clinical trials and investigated the accuracy, specificity and precision of these assays. Smith et al.^[^ ^185^ ^]^ assessed flow cytometry in clinical trials for the detection of T‐cell immune responses induced by tuberculosis vaccines and determined the specifics of this assay as a possible measurement of biomarkers. In summary, flow cytometry has been standardized and validated, and has been fully implemented as an endpoint assay in several vaccine trials. Although flow cytometry is a superior method of cell analysis, its biggest disadvantage is the high cost. Therefore, less expensive devices using the principles of flow cytometry are likely to be developed.

#### 4.3.3 Mesoscale Discovery Assays

MSD^[^ ^186^ ^]^ using an improved electrochemiluminescence detection system, provides an alternative multiplexing technology platform for the detection of protein biomarkers, especially cytokines, with high sensitivity. In contrast to the Luminex system that is in a liquid phase, the MSD detection system is performed on a solid phase. This system^[^ ^187^ ^]^ uses multi‐well plates fitted with up to ten carbon electrodes per well, with each electrode being coated with a specific capture‐antibody. The assay procedure follows that of a sandwich ELISA, with analytes captured on the electrode being detected with an analyte specific ruthenium‐conjugated secondary antibody. Upon electrochemical stimulation, the ruthenium label emits light at the surface of the electrodes, then sensitive photodetectors collect and quantitatively measure the light emitted from the microplates, allowing the concentration of the analyte to be determined relative to the particular electrode.

Due to high‐sensitivity, short assay time and the capability for multiplex detection, MSD technology has been used to detect ultralow levels of cytokines (up to five logs of linear dynamic range) performed with very low amounts of sample.^[^ ^188^ ^]^ Dabitao et al.^[^ ^189^ ^]^ compared the multiplex measurement of pro‐inflammatory cytokines (IL‐6, IL‐8, IL‐10, TNF‐*α*, IL‐12p70, and IL‐1*β*) in human serum using two commercial assays, the MSD assay and the Cytometric Bead Array (CBA) flow cytometric assay. It was demonstrated that the MSD assay provided a more reliable assessment of the pro‐inflammatory cytokines tested in the serum of healthy and HIV‐infected individuals. In serum, the MSD platform consistently quantified levels of endogenous IL‐12p70, TNF‐*α*, and IL‐10 that were undetectable by the CBA assay. Chaturvedi et al.^[^ ^190^ ^]^ developed a novel panoptic IL‐6 MSD assay for quantification of both high and low molecular weight (MW) IL‐6 with sensitivity of 9.77 pg L^−1^. The top‐performing antibody pair from 36 capture and four detection candidates was validated on the MSD platform. High MW forms of IL‐6, in size fractionated serum samples from myelodysplastic syndrome and rheumatoid arthritis patients were detected by the assay but not by a commercial kit. This panoptic IL‐6 MSD assay may be useful to evaluate total IL‐6 concentrations across normal and diseased indications for greater understanding of IL‐6 functionality and responses and/or resistance to anti‐IL‐6 therapeutics. Thus, the MSD assays have great advantages for high‐sensitive quantification of cytokines but it is expensive requiring centralized instrument and kits and is not suitable for PoC testing.

### 4.4 Mass Cytometry

In recent years, mass cytometry,^[^ ^191^, ^192^ ^]^ a fusion of two experimental platforms (flow cytometry and elemental mass spectrometry), has been increasingly used for the rapid analysis of single cells. This assay enables measurement of over 40 simultaneous cellular parameters at single‐cell resolution, significantly facilitating high‐dimensional, quantitative analysis of the effects of bioactive molecules on cell populations and thus enhancing the ability of cytometry to evaluate complex cellular systems and processes.^[^ ^192^, ^193^ ^]^ Mass cytometry utilizes rare earth metal isotopes as tags bound to antibodies, instead of fluorophores.^[^ ^194^ ^]^ Additional details regarding the basic principles and workflow of a typical mass cytometry technology platform can be found in Spitzer and Nolan^[^ ^192^ ^]^

Due to the characteristic of discrete readouts, the use of isotopes in mass cytometry as reporters increases the number of measurable parameters per cell.^[^ ^193^ ^]^ Additionally, this platform is quantitatively accurate with linear sensitivity across four orders of magnitude. Thus, the high‐dimensional mass cytometry enables simultaneous and highly sensitive measurement of multiple cytokines from innate and adaptive immune cell subsets with single‐cell granularity. For example, Baxter et al.^[^ ^194^ ^]^ developed a comprehensive mass cytometry assay for single‐analysis of immunophenotype and cytokine production in peripheral whole blood. This single‐cell proteomic approach enables simultaneous evaluation of multiple immune cell types, and detection of various cytokine perturbations in the milieu of patient specific "pathogenic" peripheral blood. The peripheral blood analysis via mass cytometry also provides a platform to identify patient‐specific dysregulated cell subsets and their abnormal cytokine production in autoimmune disease, which allows for the personalization of therapeutic options. As a consequence, specific treatment choices can be tested in vitro to assess their immunomodulation effectiveness. To achieve a single‐cell system‐level perspective of SLE immunopathogenesis, O'Gorman et al.^[^ ^195^ ^]^ performed phenotypic and functional (cytokines) characterization of pediatric SLE patients and healthy controls blood via mass cytometry to understand how cellular and molecular perturbations may drive SLE disease activity. The analysis revealed a distinct monocyte cytokine signature shared among clinically heterogeneous pediatric SLE patients. This study demonstrates the application of the mass cytometry platform for understanding immune dysregulation mechanisms in autoimmune disorders. However, this technique has some limitations^[^ ^191^ ^]^ including the strict requirement for a separate metal isotope per probe (no equivalent of forward or side scatter) and being destructive (no possibility of sorted cell recovery). The current configuration of the mass cytometer also has a limited cell transmission rate, thus requiring a higher input number of cells.

### 4.5 SomaLogic SOMAscan Assay

SomaLogic SOMAscan (slow off‐rate modified aptamer scan) assay^[^ ^196^, ^197^ ^]^ is developed for affinity‐proteomic analysis which allows simultaneous measurement and quantitation of over 1000 proteins directly in serum, other body fluids and cell lysates. This technique is based on aptamer binding. SomaLogic^[^ ^198^ ^]^ has developed slow off‐rate modified aptamers called SOMAmers which are protein recognition reagents with high binding affinities, stable chemical structures, easy production and an established selection processes. The selected target aptamers with 3D structure are modified into a “SOMAmer” that can bind proteins with high specificity and affinity.^[^ ^199^ ^]^ Protein targeted SOMAmer are collected, labeled, denatured and hybridized to DNA arrays fabricated by the complementary strand of the SOMAmers. This assay can accurately and rapidly identify and quantify multiple proteins across approximately eight logs of concentration in small sample volumes.^[^ ^196^ ^]^ Christiansson et al.^[^ ^199^ ^]^ introduced SomaLogic SOMAscan and other multiplex platforms for proteins (such as IFN‐*γ*, MCP‐1, IL‐8, IL‐6, VEGF) quantification by running pre‐ and post‐treatment plasma samples from Chronic myeloid leukemia (CML) patients. Lim et al.^[^ ^200^ ^]^ used the aptamer based SomaLogic SOMAscan assay and the multiplex bead‐based assay to identify circulating proteins predictive of response to immunotherapy in melanoma patients treated with a combination of immune checkpoint inhibitors. This study assessed the expression of four proteins (IL‐1RA, IL‐1A, TNF‐*α*, and IL‐6) and highlights significant limitations imposed by inconsistent sensitivity and specificity due to differences in the detection antibodies or aptamers utilized in these widespread biomarker discovery approaches. Furthermore, this assay is relatively expensive which limits its wide application especially in large scale clinical trials.^[^ ^196^ ^]^

## 5 Conclusions and Future Perspectives

Cytokine quantification is a highly active field of study and can provide comprehensive insights for disease diagnosis and treatment. The current techniques have successfully demonstrated their potential to provide highly sensitive and time‐efficient detection with various signal readouts either for in vitro or in vivo applications. In this review, we summarized various cytokine functions in immune and inflammatory responses and reviewed the stability of cytokines under different processing and storage conditions which is critical for accurate and reliable cytokine quantification. Furthermore, we discussed recent advances of several in vitro and in vivo cytokines assays by comparing their advantages and disadvantages. It is challenging to detect cytokines at desirable detection limits for achieving reliable outcomes, although significant efforts have been dedicated to developing cytokine assays with enhanced performance including sensitivity, biocompatibility and stability. Research in the area of cytokines quantification is still in its developing stages and we are on the way to achieve effective solutions for accurate and real‐time detection of multiple cytokines in vivo.

The take home message for cytokine quantification in clinical analyses include: 1) assay reliability: considering the short half‐life of cytokines, proper sample preparation/handling is essential to make sure that the analysis is accurate and reliable. Understanding the actual status of the cytokines and potential interferences in clinical samples is essential to develop reliable assays, 2) sensitivity: the recent CRISPR/Cas advancement in analytical science has demonstrated great potential for biosensors with super high sensitivity and specificity.^[^ ^159^, ^201^ ^]^ Due to the low abundance of cytokines, it is expected that CRISPR/Cas biosensing technology will definitely contribute to cytokine detection in clinical analysis, 3) PoC testing: PoC tests will play an important role as an essential component of clinical diagnosis, especially for monitoring responses to treatment. Microfluidic chips, particularly microfluidic paper based analytical devices have demonstrated great potential for this purpose,^[^ ^202^, ^203^ ^]^ we expect to hear more about their success in cytokine quantification, 4) multiplex detection: cytokines form complex cytokine network and multiplex cytokines assays provide comprehensive information about the role of immune activation and inflammation in the pathogenesis of multiple disease states simultaneously. Commercially available cytokine kits with high throughput and short assay times meet requirements for multiple cytokine measurements, offering tremendous assay advantages,^[^ ^204^, ^205^ ^]^ while their performance requires further improvement in terms of real‐time quantification capability, cost, and simplicity. Exploration of different signal readout modalities and development of microfluidic chips will be helpful to address this challenge, and 5) real‐time monitoring: cytokines are subject to dynamic secretion processes. Thus, real‐time analyses of cytokines are essential for the precise determination and characterization of immune states for clinical diagnosis and treatment. Real‐time cytokine detection can be realized by using structure‐switching aptamer‐based biosensors. The development of PoC devices combined with microfluidic techniques is promising for rapid and real‐time cytokine quantification.^[^ ^113^, ^206^ ^]^ There are unmet needs to develop a technology which is able to conduct measurements in real‐time with high accuracy and efficiency, and to recognize and differentiate between various cytokines simultaneously while providing output signals efficiently and rapidly in a PoC fashion. However, it is not easy to realize a technology with all these advantages. It is fair to speculate that there will be great progress regarding cytokine quantifications with the aid of new tools and discoveries in cross disciplinary fields such as nanotechnology, biotechnology, and molecular and device engineering.

## Conflict of Interest

The authors declare no conflict of interest.
